# Molecular stratification of early breast cancer identifies drug targets to drive stratified medicine 

**DOI:** 10.1038/s41523-016-0003-5

**Published:** 2017-02-15

**Authors:** Jane Bayani, Cindy Q. Yao, Mary Anne Quintayo, Fu Yan, Syed Haider, Alister D’Costa, Cassandra L. Brookes, Cornelis J. H. van de Velde, Annette Hasenburg, Dirk G. Kieback, Christos Markopoulos, Luc Dirix, Caroline Seynaeve, Daniel Rea, Paul C. Boutros, John M. S. Bartlett

**Affiliations:** 1 Ontario Institute for Cancer Research, Toronto, ON Canada; 2 Cancer Research UK Clinical Trials Unit, University of Birmingham, Birmingham, UK; 3 Leiden University Medical Center, Leiden, The Netherlands; 4 University of Mainz, Mainz, Germany; 5 Helios Medical Center, Schleswig, Germany; 6 Athens University Medical School, Athens, Greece; 7 St. Augustinus Hospital, Antwerp, Belgium; 8 Erasmus MC Cancer Institute, Rotterdam, The Netherlands; 9 University of Toronto, Toronto, Canada

## Abstract

Many women with hormone receptor-positive early breast cancer can be managed effectively with endocrine therapies alone. However, additional systemic chemotherapy treatment is necessary for others. The clinical challenges in managing high-risk women are to identify existing and novel druggable targets, and to identify those who would benefit from these therapies. Therefore, we performed mRNA abundance analysis using the Tamoxifen and Exemestane Adjuvant Multinational (TEAM) trial pathology cohort to identify a signature of residual risk following endocrine therapy and pathways that are potentially druggable. A panel of genes compiled from academic and commercial multiparametric tests as well as genes of importance to breast cancer pathogenesis was used to profile 3825 patients. A signature of 95 genes, including nodal status, was validated to stratify endocrine-treated patients into high-risk and low-risk groups based on distant relapse-free survival (DRFS; Hazard Ratio = 5.05, 95% CI 3.53–7.22, *p* = 7.51 × 10^−19^). This risk signature was also found to perform better than current multiparametric tests. When the 95-gene prognostic signature was applied to all patients in the validation cohort, including patients who received adjuvant chemotherapy, the signature remained prognostic (HR = 4.76, 95% CI 3.61-6.28, *p* = 2.53× 10^−28^). Functional gene interaction analyses identified six significant modules representing pathways involved in cell cycle control, mitosis and receptor tyrosine signaling; containing a number of genes with existing targeted therapies for use in breast or other malignancies. Thus the identification of high-risk patients using this prognostic signature has the potential to also prioritize patients for treatment with these targeted therapies.

## Genetics: Expression signature identifies high-risk patients

A gene expression signature identifies breast cancer patients who do poorly after endocrine therapy and might benefit from extra treatment. A team led by John Bartlett and Paul Boutros from the Ontario Institute for Cancer Research in Toronto, Canada, measured the activity levels of 165 genes known to be involved in breast cancer development in tumor samples from 3825 patients with early estrogen receptor-positive disease. The patients received either endocrine therapies (tamoxifen or an aromatase inhibitor) alone or additional chemotherapy as well. The researchers identified a 95-gene expression signature that, when combined with a determination of whether the cancer has spread into the lymph nodes, can help predict which patients are at high risk of disease progression, regardless of whether they received chemotherapy or not. These patients could be prioritized for additional drug therapies.

## Introduction

Despite significant improvements in the treatment of early estrogen receptor positive (ER+) breast cancer, there are ongoing clinical challenges. Targeted anti-endocrine therapies have reduced mortality over the last 30–40 years,^1, 2^ but ER+ disease, which comprises 80% of breast cancers, still leads to the majority of deaths from early breast cancer.^3^ Multiparametric gene assays are used increasingly to guide clinical treatment decisions.^4^ Most prognostic tests provide an estimate of relapse risk following the treatment for ER+ breast cancer, but still lack predictive value for novel targeted treatment options.^2, 4^ These multiparametric tests, which include OncotypeDx® (Genomic Health Inc.),^5, 6^ Prosigna^™^ (NanoString Technologies, Inc.),^7–9^ MammaPrint® (Agendia Inc.),^10, 11^ Breast Cancer Index (BioTheranostics Inc.),^12, 13^ and EndoPredict (Sividon Diagnostics GmbH),^14^ all provide broadly similar clinical utility.^15, 16^ Although each is derived from RNA abundance studies, there are surprisingly few overlapping genes between different RNA signatures.^17^ Prat et al., demonstrated in silico that combined signatures may more accurately predict outcome; leading to greater clinical significance.^18^ Nonetheless, despite a decade of development of multiple residual risk signatures, progress towards stratified or targeted medicine has not been markedly accelerated by these tests. None of the existing tests have identified actionable targets which might form the basis for the next generation of stratified medicine approaches.

Using the Tamoxifen and Exemestane Adjuvant Multinational trial (TEAM) pathology cohort,^19^ comprised of 3825 hormone-receptor positive (ER+ and/or PgR+) cases and including 477 (13%) HER2-positive cases, we have discovered and validated a novel 95-gene signature of residual risk, which has the potential to markedly improved risk stratification in the context of endocrine-treated patients. Moreover, this gene signature has also revealed potentially druggable targets, thus moving closer to the vision of stratification to targeted therapies for such high-risk patients.

## Results

The RNA abundance profiles of all genes were generated for 3825 patients. Of patients who had complete therapy information, 2549 were treated with endocrine therapies alone, while 1275 also received adjuvant chemotherapy. The endocrine-treated only patients were divided into a 576-patient training cohort (*n* = 67 events), and a 1973-patient validation cohort (*n* = 253 events), which was used for signature discovery and validation, respectively. To test the prognostic ability of the signature, which was trained and validated in the endocrine-treated patients, to patients who were treated with adjuvant chemotherapy, the signature was then modeled against all patients in the validation cohort and adjusted for adjuvant chemotherapy (*n* = 3035). The median follow-up in each cohort was 7.51 and 6.21 years, respectively. The clinical characteristics of the endocrine-treated training and validation cohorts are described in Table 1. The clinical characteristics of the entire cohort of 3825 patients are summarized in Supplementary Table 1. High tumor grade, nodal status, pathological size, and HER2 IHC status were univariately prognostic in both training and validation cohorts (Table 1 and Supplementary Table 1).

**Table 1:** Clinical characteristics of the endocrine-treated patients

	**Training**				**Validation**			
	**HR**	**95% CI**	*P* **-value**	*N*	**HR**	**95% CI**	*P* **-value**	*N*
Age (<55)	1.791	0.44–7.32	0.417	576	0.856	0.52–1.40	0.535	1974
*Nodal Status*								
0 vs. 1–3	1.372	0.81–2.33	0.240	567	1.323	0.98–1.78	0.066	1925
0 vs. 4–9	3.314	1.46–7.53	0.004		4.021	2.77–5.83	1.916 × 10^−13^	
0 vs. 10+	4.973	1.75–14.10	0.003		6.562	4.17–10.34	4.907 × 10^−16^	
*Pathological Size (Categorical)*								
≤2 vs. (>2 cm & ≤5 cm)	1.953	1.19–3.20	0.008	576	2.148	1.63–2.83	5.765 × 10^−08^	1972
≤2 vs. >5	3.096	0.94–10.17	0.063		2.755	1.75–4.33	1.117 × 10^−5^	
Pathological Size *(Continuous)*	1.163	1.06–1.27	0.001	560	1.311	1.21–1.42	9.401 × 10^−12^	1963
*Grade*								
1 vs. 2	1.835	0.56–5.99	0.315	563	1.433	0.90–2.29	0.131	1869
1 vs. 3	3.341	1.02–10.93	0.046		2.606	1.64–4.15	5.452 × 10^−5^	
HER2	2.31	1.33–4.02	0.003	564	1.835	1.32–2.55	2.745 × 10^−4^	1890

### Identification and validation of a residual risk signature following endocrine treatment

**Fig. 1:**
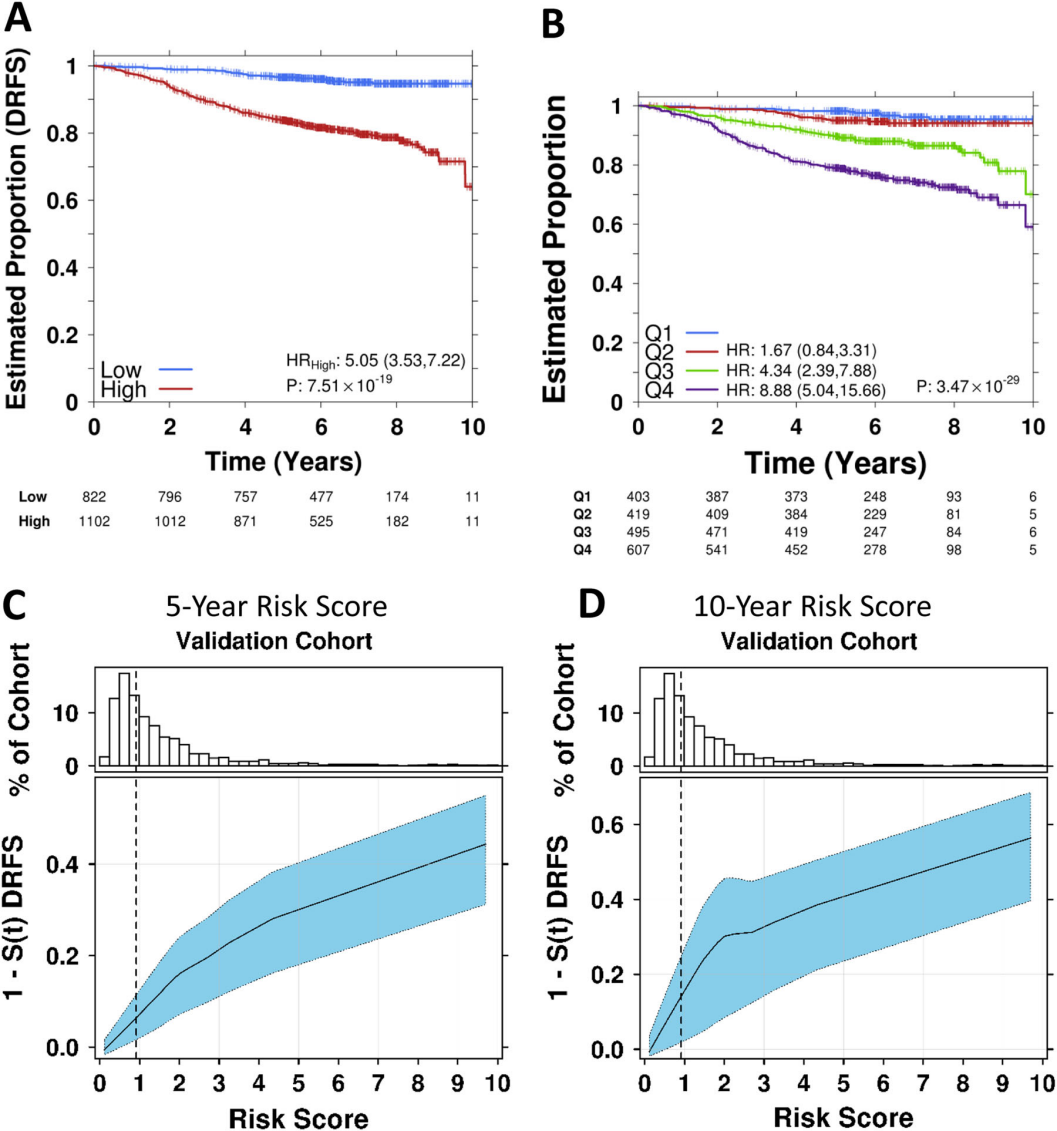
Kaplan–Meier survival plots of the 95-gene residual risk signature in the TEAM pathology cohort. **a** Survival curves based on the prognostic model including nodal status applied to the validation cohort of patients receiving only endocrine therapy. **b** Risk score estimates shown in A grouped as quartiles with each group compared against Q1. Hazard ratios were estimated using Cox proportional hazards model and significance of survival difference was estimated using the log-rank test. **c** Distribution of patient risk scores in the TEAM Validation cohort showing the predicted 5 year recurrence probabilities (*solid line*) and 95% CI (*dashed lines*) as a function of patient risk score. *Vertical dashed black line* indicates training set median risk score. **d** Distribution of patient risk scores in the TEAM Validation cohort showing the predicted 10 year recurrence probabilities (*solid line*) and 95% CI (*dashed lines*) as a function of patient risk score. *Vertical dashed black line* indicates training set median risk score

Univariate assessment of the original gene list of 165 genes identified 95 genes which were prognostically significant in the endocrine-treated only patients (Supplementary Table 2, Supplementary Fig. 1). The 95 genes were aggregated into functional modules and used to train a residual risk model. Modeling of the module dysregulation (MDS) scores (as described in the Supplementary Methods) generated from these 95 genes, with and without clinical covariates, resulted in a final refined signature that included nodal status as the only clinical covariate (Fig. 1). This risk model was found to be comparable in the training cohort when the 95-gene signature was used, without clinical covariates (HR_high_ = 4.05, 95% CI 2.25–7.3, *p* = 3.28 × 10^−6^; 10-fold cross validation ) and when clinical co-variates such as age, tumor grade, pathological tumor size, and nodal status were included (HR_high_ = 2.74, 95% CI 1.61–4.65, *p* = 2.06 × 10^−4^; 10-fold cross validation) (Supplementary Fig. 2). When dichotomized around the median and applied to the validation set, the resulting 95-gene signature was a robust predictor of DFRS following endocrine treatment (HR_high_ = 5.05, 95% CI 3.53–7.22, *p* = 7.51 × 10^−19^, Fig. 1a). As with the training set, similar results were obtained when all clinical covariates were included in the model of the validation cohort (HR_high_ = 5.56, 95% CI 3.85–8.03, *p* = 5.75 × 10^−20^, Supplementary Fig. 3). When samples were split into quartiles (Fig. 1b), the signature identified patients were at a very low risk (<5% DRFS at 10 years). The continuous risk scores from this signature were directly correlated with the likelihood of recurrence at 5(Fig. 1c) and 10-years (Fig. 1d), with a higher risk score associated with a markedly higher likelihood of a metastatic event.

### Performance of the 95-gene signature of residual risk in the presence of adjuvant chemotherapy

To determine whether the 95-gene residual signature continued to be prognostic amongst patients who also received adjuvant chemotherapy, the model was applied to all patients in the validation cohort (with and without chemotherapy), but stratified to chemotherapy (Supplementary Fig. 4A and B). The results showed that the 95-gene signature was still prognostic in this subset of patients (HR_high_ = 4.7, 95% CI 3.61-6.28, *p* = 2.53× 10^−28^). Stratifying according to adjuvant chemotherapy showed no difference in the DRFS between patients defined as low or high risk by the signature (Supplementary Fig. 4C).

### Performance of the 95-gene signature of residual risk when adjusted for HER2 status

To determine whether the 95-gene residual risk signature remained prognostic in both HER2-positive and HER2-negative patients, the model was applied to patients in the validation cohort who did not receive any additional adjuvant chemotherapy and results stratified by HER2-status (Supplementary Fig. 5). When the model was applied to all patients and stratified by HER2-status (Supplementary Fig. 5A), patients identified as low-risk by the 95-gene signature, showed no significant difference in DRFS between HER2-positive or HER-2 negative patients (*p* = 0.78). Similarly, for patients identified as high-risk, we observed no statistically significant difference in DRFS between HER2-positive or HER2-negative patients (*p* = 0.09), although we did observe that HER2-positive patients showed a trend for worse outcome. Overall, the signature can differentiate high-risk from low-risk individuals within either HER2-positive (HR = 5.17; 95% CI: 1.25–21.38; *p* = 0.023) or HER2-negative (HR = 4.75; 95% CI: 3.23–6.97; *p* = 2.01 × 10^−15^) patient subsets.

### Performance of the 95-gene signature to multiparametric tests

**Fig. 2:**
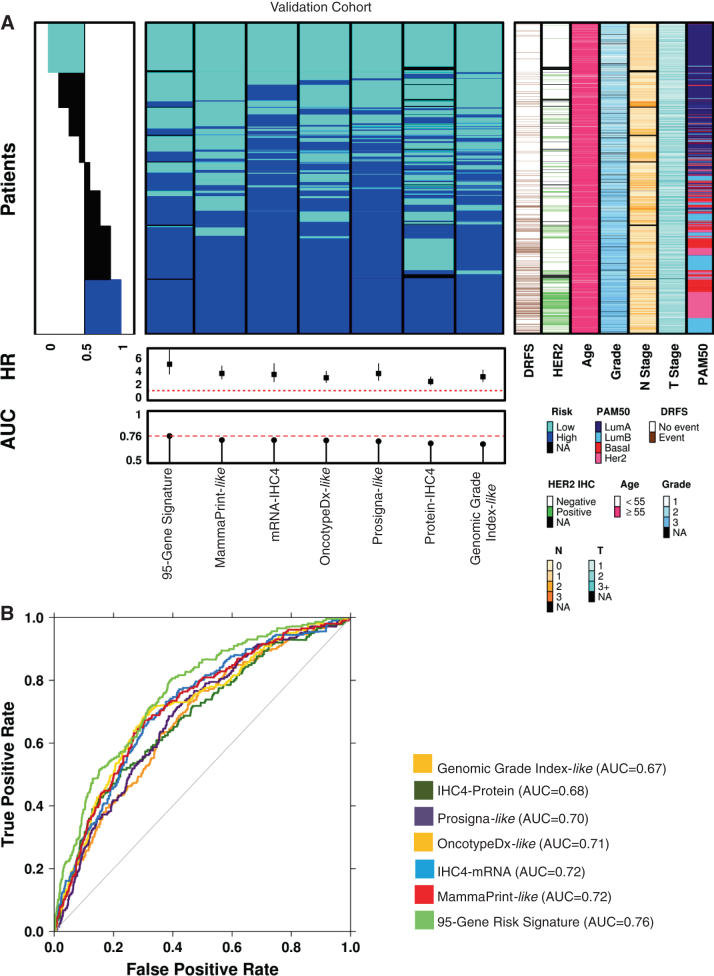
Comparison of the 95-gene residual risk signature to multi-parametric tests in the validation cohort. **a** Summary of patients assessed in the validation cohort using the 95-gene residual risk signature and other current multiparametric tests in addition to clinical covariates. Patient samples were ranked according to overall concordance, with all patients called as high-riskor low-risk, across all tests organized at the *bottom* and *top* of the heatmap, respectively. Standard clinical covariates such as HER2 status, age, grade, nodal status, stage are included. Molecular subtyping based on the PAM50/Prosigna-like test is also shown. **b** As performance indicator, area under the receiver operating characteristic (AUC) curves for each multiparametric test is also shown. All patients represented are those who only received endocrine treatment

Using the NanoString RNA abundance data, risk scores from current multiparametric test were generated and are summarized in Fig. 2a and Supplementary Table 3, along with known prognostic clinical factors. Molecular intrinsic subtyping results are also shown (Fig. 2a). While there exists a common group of high-risk and low-risk patients across all tests, there are large numbers of patients with discordant results (Fig. 2a, Supplementary Table 4). When compared to the risk scores generated based on the commercial tests (Fig. 2b, Table 2), our 95-gene signature performed better than these multiparametric tests, with an Area Under the Curve (AUC) of 0.76. The differences in AUC between the commercial tests and the 95-gene risk score were found to be statistically significant (Table 3). The summary of commercial-like risk scores across the validation cohort, in addition to the overall concordance between the tests are shown in Supplementary Tables 3 and 4, with Kaplan–Meier survival plots for each of the commercial or academic risk stratification tests shown in Supplementary Fig. 6 and described further in the Supplementary Data. Overall, each test, as recapitulated using our NanoString RNA abundance data, could discriminate with statistical significance (Supplementary Data and Supplementary Fig. 6), between patients at low or high risk for recurrence.

**Table 2:** Performance of the 95-gene residual risk signature and multiparametric tests in the validation cohort

	**HR**	**HR.95L**	**HR.95U**	*P*	*N*	**AUC**
95-Gene Signature	5.045	3.528	7.215	7.51 × 10^−19^	1924	0.76
MammaPrint-*like*	3.631	2.765	4.767	1.66 × 10^−20^	1973	0.72
Prosigna-*like*	3.49	2.592	4.699	1.75 × 10^−16^	1971	0.70
IHC4-RNA	3.475	2.346	5.148	5.11 × 10^−10^	1973	0.72
Genomic Grade Index-*like*	3.118	2.341	4.153	7.51 × 10^−15^	1973	0.67
OncotypeDX-*like*	2.969	2.232	3.948	7.37 × 10^−14^	1973	0.71
IHC4-Protein	2.398	1.851	3.108	3.72 × 10^−11^	1855	0.68

**Table 3:** Statistical Differences in AUC between multiparametric tests and the 95-gene residual risk signature

	**Genomic Grade Index**—*like*	**IHC4-Protein**	**Prosigna-** *like*	**OncotypeDX-** *like*	**IHC4-RNA**	**MammaPrint**—*like*
IHC4-Protein	6.88 × 10^−1^					
Prosigna-*like*	3.53 × 10^−1^	8.81 × 10^−1^				
OncotypeDX-*like*	2.04 × 10^−2^	8.01 × 10^−2^	8.84 × 10^−2^			
IHC4-RNA	4.16 × 10^−3^	4.28 × 10^−2^	2.23 × 10^−2^	8.11 × 10^−1^		
MammaPrint-*like*	2.21 × 10^−3^	5.78 × 10^−2^	1.21 × 10^−2^	7.81 × 10^−1^	9.50 × 10^−1^	
95-Gene Signature	2.83 × 10^−9^	4.02 × 10^−5^	3.02 × 10^−8^	5.10 × 10^−3^	4.25 × 10^−3^	2.98 × 10^−3^

### Identification of druggable targets in the 95-Gene signature and implications for stratified precision medicine

**Fig. 3:**
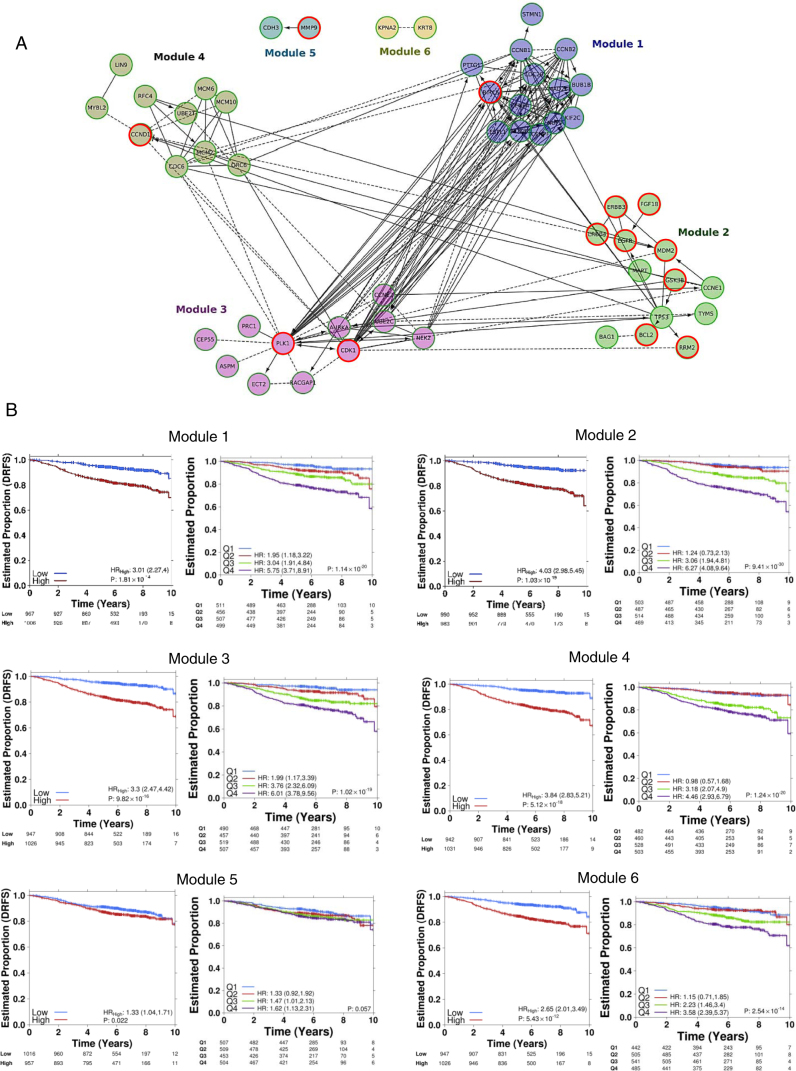
Signaling modules within the 95-gene residual risk signature. **a** Summary of REACTOME interactions amongst the genes of the 95-gene residual risk signature. Six major interaction modules comprising 52 genes were identified from the 95-gene residual risk signature. Relationships between genes, between and within modules, are shown by *connecting lines*. *Solid lines* with *arrows* indicate known and direct positive relationships. *Solid lines* ending in a *perpendicular line* indicate a known negative regulatory relationship. *Dotted lines* indicate relationships linked by other genes. Genes with *red circles* indicate gene targets for which there are known targeted therapies or at phase II/III development based on the Integrity compound search tool (Thompson Reuters) and ClinicalTrials.gov (https://clinicaltrials.gov/). **b** Kaplan–Meier survival curves (*left*) for each module are shown, and representing the validation cohort. To the *right* of each Kaplan–Meier curve are risk score estimates grouped as quartiles with each group compared against Q1. Hazard ratios were estimated using Cox proportional hazards model and significance of survival difference was estimated using the log-rank test. All patients represented are those who only received endocrine treatment

Six significant network modules were identified using the Reactome Functional Interaction (FI) tool, comprising 52 of 95 genes in the signature (Fig. 3a, Table 4). Modules 1, 3, and 4 included genes involved in mitosis (FDR < 5.0 × 10^−4^), cell cycle (FDR < 3.33 × 10^−4^), as well as pathways associated with cell cycle checkpoints (FDR = 0.0001). Module 2 included genes and pathways involved in receptor-tyrosine signaling including Erb-Receptor (ERBB) pathway signaling (FDR < 6.66 × 10^−5^), PI3K-AKT signaling (FDR < 8.33 × 10^−5^), p53 signaling (FDR < 5.00 × 10^−4^), and apoptosis (FDR = 0.00479). Normalized expression for the individual genes (Supplementary Table 5) within the modules showed that all genes within Modules 1, 3, and 4 were more highly expressed among patients classed as high-risk (Supplementary Table 5; Wilcoxon rank-sum test). These differences were found to be statistically significant (Supplementary Table 5). As individual modules, they were statistically significant predictors of outcome (Fig. 3b). Though not statistically significant, a higher AUC was observed when using all 95 genes together as a residual risk signature set and hence was carried over as the final list (HR_high_ = 5.05, 95% CI 3.53–7.22, *p* = 7.51 × 10^−19^). Module 1, comprised of genes largely associated with mitosis and regulation of the cell cycle such as BIRC5, BUB1B, CCNB1, and PTTG1 (Table 4); could classify patients in the validation cohort as low-risk or high-risk (HR_high_ = 3.01, 95% CI 2.27-4.0, *p* = 1.81 × 10^−14^). Similarly, genes from Modules 3 and 4, including Aurora Kinase A, CDK1, CCND1, CCNE2, CDC6, and PLK1, classified patients in low-risk and high-risk categories: HR_high_ = 3.3, 95% CI 2.47–4.42, *p* = 9.82 × 10^−16^ and HR_high_ = 3.84, 95% CI 2.83–5.21, *p* = 5.12 × 10^−18^, respectively (Fig. 3b). Normalized RNA abundance within Module 2 was mixed (Supplementary Table 5), with some showing decreased expression among high-risk patients (i.e. TP53 and BCL2), and others showing increased expression (i.e. CCNE1 and RRM2); but when modeled as a group, Module 2 could also identify patients with worse prognosis (HR_high_ = 4.03, 95% CI 2.98–5.45, *p* = 1.03 × 10^−19^). Finally, Module 5, comprising of CDH3 and MMP9 was also a significant predictor of DRFS (HR_high_ = 1.33, 95% CI 1.04–1.71, *p* = 0.022), as well as Module 6 comprising two genes; KPNA2 and KRT8 (HR_high_ = 2.65, 95% CI 2.01–3.49, *p* = 5.43 × 10^−12^).

**Table 4:** Summary of pathway modules comprising the 95-gene residual risk signature

**Module**	**Gene List**	**Pathways in Modules**	**Putative Targeted Therapy** ^**a**^ **(Gene Target)**
**1**	BIRC5 BUB1B CCNB1 CCNB2 CDC20 CENPA CENPF ESPL1 KIF2C MAD2L1 NDC80 NUF2 PTTG1 STMN1	Mitotic Metaphase and Anaphase, Mitotic Prometaphase, Cell cycle, Mitotic G2-G2/M phases, Aurora A and B signaling, FOXM1 transcription factor network, Oocyte meiosis, APC/C-mediated degradation of cell cycle proteins, PLK1 signaling events, Cell Cycle Checkpoints,	Gataparsen (BIRC5)
**2**	BAG1 BCL2, CCNE1 EGFR, ERBB3 ERBB4 FGF18 GSK3B MAPT MDM2 RRM2 TP53 TYMS	p53 signaling pathway, ERBB-family signaling, PIK3CA-AKT signaling, Aurora A signaling, PLK signaling, cell-cycle checkpoints, apoptotic signaling. AKT-signaling, FGFR signaling, PDGF signaling	Oblimersen Sodium (BCL2), Venetoclax (BCL2), Obatoclax Mesylate (BCL2), Navitoclax (BCL2), Patritumab (ERBB3), Sapitinib (ERBB3), Afatinib (ERBB4), Neratinib (ERBB4), Dacomitinib (ERBB4), Gefitinib (EGFR), Erlotinib (EGFR), Lapatinib (EGFR), Pan-FGFR inhibitor (AP24534, FGF18)
**3**	ASPM AURKA CCNE2 CDK1 CEP5 ECT2 NEK2 PLK1 PRC1 RACGAP1 UBE2C	PLK1 signaling, Cell cycle checkpoints, Mitotic telophase and cytokinesis, Mitotic telophase and anaphase, FOXM1 transcription	Diniciclib (CDK1), Rigosertib sodium (PLK1), Volasertib (PLK1)
**4**	CCND1 CDC6 LIN9 MCM10 MCM2 MCM6 MYBL2 ORC6, RFC4 UBE2T	S-phase, Regulation of DNA replication, Cell cycle, p53 signaling, M/G1 transition	Palbociclib (CCND1)
**5**	CDH3 MMP9	Alzheimer disease-presenilin pathway, role of ran in mitotic spindle regulation	
**6**	KPNA2 KRT8	role of ran in mitotic spindle regulation, Regulation of cytoplasmic and nuclear SMAD2/3 signaling	

Pathways chosen with False Discovery Rate (FDR) *p* < 0.001

^a^Compound search conducted using Thomson Reuters IntegritySM and ClinicalTrials.gov (https://clinicaltrials.gov/)

Using the Integrity Compound Search (Thomson Reuters) for the genes within these modules, a number of targeted compounds were identified as being currently used in the clinic for treatment of breast cancer or other neoplasms; or in phase II and/or phase III development (https://clinicaltrials.gov/) (Fig. 3, Supplementary Table 6). Among these compounds, a number have potential for stratified use in the early luminal breast cancer setting (Table 4) for those deemed high-risk by our classifier. Therefore, these compounds hold potential for repurposing targeted therapies to early luminal breast cancers (Supplementary Fig. 7).

## Discussion

Relapse following endocrine treatment remains a significant clinical challenge, as more women die following treatment for ER+ disease than for any other breast cancer subtype.^3^ Therefore, there is an ongoing need to identify women who are at risk for relapse following anti-endocrine therapy. More importantly, simultaneously identifying targets for future therapeutic intervention and the means to effectively stratify women to such targeted therapies will improve the clinical management of these patients, and potentially reducing their overtreatment, or conversely identifying patients who may be currently undertreated. Using 3825 patients from the TEAM pathology cohort, we are able to derive a signature that both significantly improves risk stratification and identifies genes for which there are drugs currently in use, or under evaluation (https://clinicaltrials.gov/) in other malignancies. These patients could potentially be matched to the specific functional modules within this 95 gene signature (Table 4, Supplementary Table 6). As alluded to by the prognostic capacity of the individual modules (Fig. 3), this approach has the potential to better stratify patients to existing targeted therapies based on the molecular drivers of their cancer, and/or to novel/putative targets for in vitro validation studies (Supplementary Fig. 7). Despite the fact that our commercial risk score and subtype classification was derived based on NanoString RNA abundance profiling, our work confirmed the recent findings of the UK-OPTIMA prelim trial^17^ that most current breast cancer multiparametric risk tests provide broadly equivalent risk information for a population of women with ER+ breast cancers (Supplementary Fig. 6), but can exhibit discordance between tests at the individual patient level (Fig. 2, Supplementary Tables 3 and 4). However, while efforts are being made to identify those patients who may be sensitive to current standard cytotoxic chemotherapies, we must also look to the promise of targeted therapies against the molecular drivers of these high-risk patients as revealed by the genes in the signature.

While current multiparametric tests can identify those who may benefit from current adjuvant chemotherapy regimens, none of these tests predicts response to a drug-specific chemotherapy. This challenge is hampered by the identification of driver pathways in addition to the complexities of both global and individual chemotherapeutic response. Using the information generated by this data we envision a model for future prospective clinical trial design through the examination and validation of the drugs targeting the gene modules comprising the 95-gene signature (Supplementary Fig. 7, Supplementary Table 6). In this way, genes associated with the G2/M checkpoint, as identified in Module 1, such as BIRC5 (Survivin), could be targeted. Indeed YM155, a Survivin suppressor, was recently evaluated in the metastatic breast cancer setting in combination with docetaxel in a phase II, multicenter, open-label, 2-arm study.^20^ However, the lack of up-front patient stratification for YM155 benefit likely contributed to the finding of no significant benefit in its addition to docetaxel, thus obscuring the potential benefit of targeting this pathway. While known to be overexpressed in breast cancers, the relatively higher expression of BIRC5 observed among our high-risk patients (*q* = 1.37× 10^−178^) (Supplementary Table 5) suggests there is a tipping point of mRNA abundance leading to increased risk. We observed that all genes within Modules 1, 3, and 4 showed a higher expression among patients at higher risk for relapse which were statistically significant (Supplementary Table 5), reflecting the prominent role of cell cycle and proliferation in breast cancer pathogenesis. We see that Module 3 is characterized by pathways involving late mitotic events. The overexpression of CDK1 offers a theranostic target, with the use of Dinacilib or similar molecules, currently under evaluation in phase III trials (Supplementary Table 6). Regaining cell cycle and mitotic checkpoint control is another attractive mechanism for directed therapies, with theranostic targets such as PLK1 (Fig. 3), being treated with inhibitors in the preclinical and clinical setting.^21, 22^ The regulation of S-phase and DNA replication pathways of Module 4, including CCND1, supports the potential stratification of patients to Palbociclib or other CDK inhibitors (Supplementary Table 6). Findings for the PALOMA-1 trial^23^ resulted in approval for Palbociclib (CDK4/6 inhibitor) in combination with Letrozole in the metatstatic breast cancer setting; paving the way for the randomization of high-risk patients with ER+/HER2-cancer and residual disease, in the PENELOPE-B trial. While promising in the late and metastatic setting, CDK inhibitor use in the early breast cancer setting has not yet been adequately assessed, nor is there a validated method to stratify patients who would most benefit from this treatment. Interestingly, recent in vitro evidence of synergy between palbociclib with tamoxifen showed resensitization to tamoxifen in ER-resistant cell lines,^24^ suggesting that the identification of those who may be ER-resistant, could experience greater benefit with the use combined use of endocrine therapy and a CDK inhibitor. Genes of Module 2 are characterized by receptor tyrosine kinase signaling, apoptosis and control of the cell cycle have druggable targets among the members of ERBB-family of genes. Anti-HER therapies are effective in ERBB2/HER2-positive patients, but crosstalk between other members of the Epidermal Growth Factor Receptor (EGFR)/ERBB family suggest the aberrant expression of members aside from ERBB2/HER2 could justify their use in the absence of HER2 amplification. EGFR inhibitors such as gefitinib and laptinib have shown efficacy in other malignancies, but only moderate success in breast cancer, suggesting that an improved method of patient selection is required to identify those who would benefit the most. Interestingly, 296/342 (86.5%) HER2-enriched-like patients were identified as high-risk by our classifier. However, with 33.8% of HER2-enriched-like patients possessing confirmed HER2 gene amplification or protein over-expression, these results suggest some patients may benefit from therapy targeting the ERBB-family and associated pathways. In fact, the 95-gene signature was still prognostic irrespective of ERBB2/HER2-status, in this population of patients that pre-dates the use of anti-ERBB2/HER2 therapies (Supplementary Fig. 5). Moreover, while ERBB3 and ERBB4 were found to be univariately prognostic and part of the final signature, ERBB2/HER2 expression was not (Supplementary Table 2). This data would suggest that in current clinical practice, a number of our ERBB2/HER2-positive, low-risk patients would have received anti-ERBB2/HER2 therapies, resulting with an outcome potentially no better than ERBB2/HER2-negative patients. With respect to high-risk patients who were also ERBB2/HER2-positive, anti-ERBB2/HER2 treatment would have some benefit to a subset in this group, but it is clear that there are other molecular drivers of recurrence in this high-risk population. Downstream pathways of ERBB, like PIK3/AKT/mTOR, which was identified as a significant pathway by our analyses, supports the potential use of everolimus in patients identified as high-risk.^25, 26^ Interestingly, 278/352 (78.9%) of patients identified as Basal-like were classified as high-risk by our gene signature despite being clinically classified as ER+; highlighting the need to recognize the importance of molecular heterogeneity among the hormone receptor-positive cancers, and the implications for novel treatment.

We have demonstrated that a novel 95-gene signature of residual risk, which integrates nodal status, has significantly better clinical utility for early recurrence than the currently available multiparametric tests. The signature appears to remain prognostic for later recurrence, though confirmation of its prognostic ability should be evaluated upon longer follow-up. Unlike these tests, modular analysis of the genes in the signature, have identified several genes and pathways suitable for therapeutic intervention among the high-risk patients. While the clinical potential of novel therapeutic targets identified in this study are being investigated,^27^ both additional independent clinical validation of the signature and preclinical validation of the effects of targeting these pathways are required prior to implementation of this approach in a clinical trial. It is clear, however, that there is a need for significant improvement in the targeted selection of patients suitable for new therapies, rather than the randomization of all-comers in future clinical trial design. As we have demonstrated, hormone-receptor positive cancers are molecularly heterogeneous, thus requiring novel treatment strategies (Fig. 2a and Supplementary Table 3, Supplementary Table 6). Clearly a multiparametric gene signature, as we have shown here is one means of selection, but improved stratification must also include the integration of gene mutational and copy-number status. Therefore we propose that in order to improve the clinical management of women with early hormone-receptor positive breast cancer, future clinical trial design requires a multiparametric test that not only improves identification of high-risk patients, but also improves the selection of patients to existing therapeutics targeting key genes/pathways that underlies the signature.

## Materials and methods

The TEAM trial was a multinational, open-label, phase III trial in which postmenopausal women with hormone receptor-positive^19^ early breast cancer were randomly assigned to receive exemestane (25 mg) once daily, or tamoxifen (20 mg) once daily for the first 2.5–3 years; followed by exemestane (25 mg) (totaling 5 years of treatment) (Supplementary Fig. 8). Hormone-receptor (ER and PgR) and HER2 status by immunohistochemistry were locally assessed for entry into the trial and then centrally confirmed,^28^ and HER2 status was confirmed by immunohistochemistry and fluorescence in situ hybridization.^29^ All assessment was performed according to American Society of Clinical Oncology (ASCO)/College of American Physicians (CAP)/ guidelines.^30–32^ None of the patients received anti-HER2 therapy. This study complied with the Declaration of Helsinki, Institutional Ethics Committee Guidelines, and the International Conference on Harmonisation and Good Clinical Practice guidelines. All patients provided informed consent. DRFS was defined as time from randomization to distant relapse or death from breast cancer.^19^

The TEAM trial included a pathology research study comprised of 4736 patients from five countries with an average clinical follow-up of 6.86 years. Power analysis was performed to confirm the study size had 88.6 and 100% power to detect a HR of at least 3.0 in the training and validation cohorts respectively (Supplementary Figure 8B). RNA was available and successfully assayed from 3825 samples. Patients from the UK cohort were assigned as the training cohort (*n* = 790); while the remaining patients from Germany, Belgium, Netherlands, and Greece comprised a fully-independent validation cohort (*n* = 3035). All patients were assayed for mRNA abundance (Supplementary Fig. 8C). To identify a signature of residual risk following endocrine treatment only, the main analyses excluded those patients who received neo-adjuvant and adjuvant chemotherapy. However, analyses of patients who also received adjuvant chemotherapy using the signature trained in the absence of chemotherapy are discussed and included in the Supplementary Results.

### RNA extraction and expression profiling

Five 4 µm formalin-fixed paraffin-embedded (FFPE) sections per case were deparaffinised, tumor areas were macro-dissected and RNA extracted using the Ambion® Recoverall^™^ Total Nucleic Acid Isolation Kit-RNA extraction protocol (Life Technologies^TM^, Ontario, Canada). RNA aliquots were quantified using a Nanodrop-8000 spectrophometer (Delaware, USA). All 3825 RNAs extracted from the TEAM pathology cohort were successfully assayed. Probes for each gene were designed and synthesized at NanoString® Technologies (Seattle, Washington, USA); and 250 ng of RNA for each sample were hybridized, processed and analyzed using the NanoString® nCounter® Analysis System, according to NanoString® Technologies protocols.

### mRNA abundance analysis and survival modeling

Raw mRNA abundance count data were pre-processed using the NanoStringNorm R package^33^ (v1.1.19) using normalization factors derived from the geometric mean of the top expressing 75 genes. Samples with RNA content |z-score| >6 were flagged and removed as outliers. To assess the performance of the chosen normalization method in this cohort, a combination of 252 preprocessing methods were evaluated. Firstly, each preprocessing method was ranked based on their ability to maximize Euclidean distance of ERBB2 mRNA abundance between HER2-positive and HER2-negative samples. The process was repeated for one million random subsets of HER2-positive and HER2-negative samples for each of the preprocessing schemes. Fifteen replicates of an RNA pool extracted from selected anonymized FFPE breast tumor samples were profiled across multiple batches; and preprocessing methods were ranked based on the inter-replicate variation. A mixed effects linear model was fit and residual estimates were used as an estimate of inter-batch variation (nlme v3.1–117). Cumulative ranks based on these two criteria were calculated using RankProduct and the method chosen was amongst the top 10 preprocessing methods in rank product (Supplementary Data, Supplementary Fig. 9).

Univariate survival analysis of preprocessed mRNA abundance data was performed by median-dichotomizing patients into high-expressionand low-expression groups. Clinical variable age was modeled as binary (dichotomized around age 55), while grade and nodal status were modeled as ordinal variables, and pathological size was modeled as a continuous variable.

### Network-based signature derivation

Feature-selection of genes was first performed based on univariate Cox proportional hazards modeling in the endocrine-treated only training cohort; those with *p* < 0.25 were retained. These retained genes were used to calculate a “module-dysregulation score” (MDS; Supplementary Data). A multivariate Cox proportional hazards model was then fit on MDSs, along with clinical covariates (age, grade, pathological size and nodal status); a stepwise backward selection approach using Akaike Information Criterion was performed to refine the multivariate model. The final selected model was trained in the training cohort and validated in the fully independent validation cohort (Table 1). DRFS truncated to 10 years was used as an end-point. Recurrence probabilities were estimated as described in Supplementary Data. All survival modeling was performed on DRFS, in the R statistical environment with the survival package (v2.37–4). Model performances were evaluated through area under the receiver operating characteristic (ROC) curve (AUC, Supplementary Data).

### Derivation of commercially-based and academically-based risk stratification scores

The derivation of similar risk classifications using genes comprising the following multi-parametric tests OncotypeDx® (Genomic Health Inc.),^5, 6^ Prosigna^™^(NanoString Technologies, Inc.),^7–9^ MammaPrint® (Agendia Inc.),^10, 11^ Genomic Grade Index^34^; in addition to IHC4^35, 36^ are described previously by Prat et al.,^18^ and in the Supplementary Data and Supplementary Table 7.

### Pathway analyses using reactome

The final gene list was loaded into the Cytoscape Reactome FI plugin in Cytoscape (v3.0.2). Symbols were loaded as a gene set with the 2013 version of the FI network. A FI network was constructed with FI annotations and no linker genes. Spectral clustering and pathway enrichment were computed for each module using the Reactome FI plugin functions.

## Electronic supplementary material

Supplementary Data

Supplementary Figure 1

Supplementary Figure 2

Supplementary Figure 3

Supplementary Figure 4

Supplementary Figure 5

Supplementary Figure 6

Supplementary Figure 7

Supplementary Figure 8

Supplementary Figure 9
