# Supplementary material for: Molecular stratification of early breast cancer identifies drug targets to drive stratified medicine
Source: NPJ Breast Cancer. 2017 Feb 15;3:3. doi: 10.1038/s41523-016-0003-5 (PMC5445616; doi:10.1038/s41523-016-0003-5)
Supplement: Supplementary file 1 — Supplementary Data [file 41523_2016_3_MOESM1_ESM.docx]

***SUPPLEMENTARY DATA***

***METHODS***

**TEAM Cohort Power Calculations**

To evaluate whether there was sufficient power to develop prognostic markers in this study, power calculations were performed for both endocrine-only cohort, as well as the endocrine + adjuvant chemotherapy cohort; the complete TEAM cohort (n = 2549 and events = 320; n = 3,825 and events = 507); and for each of the training (n = 576 and events = 67; n = 790 and events = 106) and validation (n = 1973 and events = 253; n = 3,035 and events = 431) subsets separately. Assuming equal-sized patient groups, power estimates representing the likelihood of observing a specific HR against the above-mentioned event numbers were derived using the formula ^1^:

$$z_{power}=\frac{\sqrt{E}\times\ln(HR)}{2}-z(1-\frac{\alpha}{2})$$

where *E* represents the total number of events (DRFS) and *α* represents the significance level which was set to 10^-3^ to represent multiple testing adjustment. *z_power ­_*was calculated for HR ranging from 1 to 3 with steps of 0.01^2^ (Haider *et al. submitted*).

**mRNA Abundance Data Processing**

Raw mRNA abundance counts were loaded and preprocessed using R package NanoStringNorm ^3^ (v1.1.19). A total of 252 combinations of preprocessing methods were evaluated: spanning normalization methods that make use of six positive controls, eight negative controls and eight housekeeping genes (RPLP0, TFRC, MRPL19, SF3A1, GAPDH, PSMC4, ACTB, and GUS) followed by global normalization (Supplementary Figure 9). To identify the most optimal preprocessing parameters, two criteria were assessed. Firstly, each test method was ranked based on their ability to maximize the Euclidean distance of ERBB2 mRNA abundance between HER2-positive and HER2-negative samples. The process was repeated for 1 million random subsets of HER2-positive and HER2-negative samples for each of the preprocessing schemes. Secondly, each preprocessing method was evaluated and ranked based on their ability to minimize inter-batch variation by using 15 replicates of an RNA pool extracted from 5 randomly selected anonymized FFPE breast tumor samples. A mixed effects linear model was performed and residual estimates were used as a measure of inter-batch variation (R package: nlme v3.1-117). Finally, cumulative ranks were estimated based on RankProduct ^4^ of the two metrics. The final selection of an optimal pre-processing method was chosen based on the rank product, which normalizes the raw counts to the geometric mean derived from the top 75 expressing genes. Fourteen samples were removed as being potential outliers (having RNA content |z-score| > 5 or low inter-array correlations). Fourteen samples were run in duplicates, and their raw counts were averaged and subsequently treated as a single sample prior to normalization.

**Module Dysregulation Score**

Module dysregulation scores (MDS) were calculated using the following process Haider *et al.*, submitted): 1) weights (β) of all evaluated genes were calculated by fitting a univariate Cox proportional hazards model based on the Training cohort only; and 2) these weights were then multiplied to the scaled mRNA abundance levels to estimate per-patient module dysregulation score:

$$MDS=\sum_{i=1}^{n} \beta X_{i}$$

Here, n represents the number of genes in a given module and X_i_ represents the scaled (z-score) abundance of gene i. MDS for patients in the Validation cohort were generated using parameters estimated through the Training cohort.

**Recurrence Probability**

Recurrence probabilities at 5- and 10-years were estimated by splitting the predicted risk-scores in 25 equal bins. For each bin, recurrence probability R(t) was calculated as 1-S(t), where S(t) is the Kaplan-Meier survival estimate at year 5 or year 10. A local polynomial regression was used to smooth the R(t) estimates of these 25 bin. The predicted estimates were then plotted against the median risk score of each group except the first and last group, where the lowest risk score and 99th percentile were used, respectively. All survival modelling was performed in the R statistical environment (R package: survival v 2.38-3).

**Model Evaluation**

Performance of survival models was evaluated using the area under the receiver operating characteristic (ROC) curve. A permutation analysis was performed to evaluate the significance of AUC differences across the different models (scores were shuffled 10,000 times while preserving the order of the survival objects).

**Derivation of Academically- and Commercially-based Risk Stratification Scores**

*mRNA-IHC4 Risk Score:* IHC4-protein model risk scores were calculated as described ^5, 6^ and adjusted for clinical covariates. ER10 scores were calculated by dividing ER histoscores by 30 and PgR10 scores were calculated by dividing the percent PgR staining by 10. A 10-fold cross validation approach was used to train the model and generate IHC4 RNA risk scores. An mRNA-IHC4 model was trained on mRNA abundance profiles of ESR1, PGR, ERBB2 and MKI67 in the training cohort using multivariate Cox proportional hazards modelling (Supplementary Table 7). Model predictions (continuous risk scores) were grouped into quartiles and analysed using Kaplan-Meier analysis and multivariate Cox proportional hazards model adjusted for clinical variables as above.

*OncotypeDX-Like Recurrence Score:* Data from the 16 test genes were normalized as previously described ^7^ and NanoString intensity values log2 transformed to fit the 0-15 measurement range from the original publication. Unscaled recurrence scores were then calculated based on: RS_U_ = + 0.47 × GRB7 group score – 0.34 × ER group score + 1.04 × proliferation group score + 0.10 × invasion group score + 0.05 × CD68 – 0.08 × GSTM1 – 0.07 × BAG1; and finally the scores are scaled as previously described ^7^ Patients were then classified into high or low outcome groups based on a recurrence score of above or below 25, respectively; and modeled for DRFS.

*Prosigna-Like Subtyping and Risk of Recurrence Score:* Samples were scored based on the method outlined by Parker *et al*.^8^ and trained in the context of ER-positivity, using the 50 genes of the PAM50 gene list ^9-11^. The “normal-like”
 subgroup was removed from the final subtyping classification. R scripts were obtained from the supplementary files ^8^ and scores were generated which were then modelled against DRFS.

*MammaPrint-Like Risk Score:* Samples were scored based on the gene70 function of the genefu R package (v1.14.0). Derivation of low and high-risk categories were modelled according to van de Vijver *et al*. ^12^ and outcome based on DRFS.

*Genomic Grade Index-Like Risk Modelling:* Samples were scored based on the procedure outlined in Toussaint *et al.*^13^ using MYBL2, KPNA2, CDC2 and CDC20*.* Expression data was used to calculate average expression housekeeping genes (GUS, TBP, RPLPO and TFRC), which was used to normalize the expression of the four genes used to determine the GGI score. Patients were classified into low or high risk groups and modelled for DRFS.

***SUPPLEMENTARY RESULTS***

***Performance of the 95-gene Residual Risk Signature and Multi-Parametric Tests:***

The composition of the gene list enabled the derivation of similar risk classifications representing a number of commercial and academic residual risk stratification tests (Figure 2). Kaplan Meier survival plots for each of the commercial and academic tests across the validation cohort showed statistically significant results consistent with the performance for those multi-parametric tests (Supplementary Figure 6, Supplementary Tables 3 and 4). In direct comparisons, the 95-gene residual-risk classifier produced an area under the curve (AUC) of 0.76, which performed significantly better than other multiparametric test results (Tables 2 and 3). The next best performing classifiers were the MammaPrint-*like* results (AUC=0.72), OncotypeDx-*like* (AUC=0.71), mRNA-IHC4 (AUC = 0.72), Prosigna-*like* (AUC=0.70), protein-IHC4 (AUC=0.68), and the Genomic Grade Index-*like* results (AUC = 0.67). The 95-gene signature performed significantly better than the Genomic Grade Index-*like* (2.83 x10^-9^) and Prosigna-*like* (p=3.02 x10^-8^) results; while also performing better than OncotypeDx-*like* (p=5.10 x10^-3^) and MammaPrint-*like* (p=2.98 x10^-3^) test results generated by our NanoString expression data. Concordance between tests is shown in Supplementary Table 5 with the 95-gene signature exhibiting greater concordance with MammaPrint-*like* and Prosigna-*like* results.

***Prosigna-like Risk of Recurrence Scores and Molecular Subtyping:*** Using the genes comprising the Prosigna test, 1971 patients across the endocrine-only treated validation cohort n=194 were identified as being low risk; n=744 were identified as having intermediate risk; and n=1033 identified as being high risk (Supplementary Table 3, Supplementary Figure 6). The observed DRFS of the validation cohort confirmed previous studies, showing those identified as low- and intermediate-risk experiencing a longer DRFS over high-risk patients (p=1.65 x 10^-17^) (Supplementary Figure 6). Combining low- and intermediate-risk patients resulted in an HR_high_ of 3.49, 95% CI 2.59-4.7, p=1.75 x10^-16^ (Supplementary Figure 6). Molecular subtyping identified 777 (39.3%) patients as Luminal A, 502 patients as Luminal B (25.4%), 352 (17.8%) patients as possessing a Basal-*like* molecular signature; and 342 (17.5%) patients with a HER2 enriched-*like* molecular signature (Supplementary Table 3). Of those patients who were identified as being HER2 enriched-*like*, and for whom information was available for HER2 status, 113/334 (33.8%) were found to be amplified for HER2 or positive for HER2 overexpression by IHC, while the remaining 221 (66.2%) were negative for gene amplification of protein expression. Differences between the DRFS across molecular subtypes showed a longer DRFS was experienced by Luminal A-classified patients (Supplementary Figure 6), over Luminal B-classified patients; and similarly, Basal-*like* patients experienced a similar DRFS at 8 years with HER2 enriched-like cases also experiencing shorter DRFS (p=8.88 x10^-23^) (Supplementary Figure 6).

***OncotypeDx-like Risk Score:*** OncotypeDx-*like* risk scores were generated according to Paik *et al.* ^7^. In keeping with the cut-off used in the TAILORx study ^14, 15^, patients were dichotomized into low- or high-risk groups using a risk score of 25 as the cut-off (Supplementary Figure 6), identifying 936 (47.4%) were deemed low-risk and 1037 patients (52.6 %) deemed high-risk. A shorter DRFS was shown for patients with risk scores greater than 25 than those with lower risk scores (HR_high_ = 2.97, 95% CI 2.23-3.95, p=7.37 x10^-14^).

***MammaPrint-like Risk Assessment***: MammaPrint-*like* risk assessment identified 1125 (57.0%) patients across the endocrine only treated cohort identified as being low risk; and 848 (43%) identified as being high risk. DRFS was longer for low-risk patients and shorter for MammaPrint-*like* high-risk patients (HR_high_ = 3.63, 95% CI 2.77-4.77, p=1.66 x10^-20^, Supplementary Figure 6).

***Genomic Grade Index-Like Risk Modelling:*** When patients were stratified according to the Genomic Grade Index using 995 patients (50.4%) were identified as low risk with the remaining 1018 patients (49.6%) deemed high risk (HR_high_=3.12, 95% CI 2.34-4.15, p=7.51 x10^-15^) Supplementary Figure 6).

***IHC4-mRNA Risk Assessment:*** Conversion of the protein-based residual risk classifier, IHC4 using the expression values of ER, PgR, Ki67 and HER2 within the code set resulted in 569 (28.8%) patients identified as low-risk and 1404 (71.2%) patients identified as high-risk within the endocrine-only treated patients. DFRS for endocrine-only treated patients deemed low-risk by IHC4-mRNA was longer than those deemed as high-risk (HR_high_=3.48, 95% CI 2.35-5.15, p=5.11 x10^-10^, Supplementary Figure 6). Indeed the use of mRNA was comparable in HR to IHC4 using the immunohistochemical results according to the original report, (HR_high_=2.4, 95% CI 1.85-3.11, p=3.72 x10^-11^, Supplementary Figure 6).

**Supplementary Figure Legends**

***Supplementary Figure 1: Univariate Results of Genes Comprising the 95-Gene Residual Risk Signature***

Heatmap showing the normalized and scaled mRNA abundance profiles of the 95 genes comprising the final residual risk signature, in the training cohort of endocrine-treated patients only.

***Supplementary Figure 2: Kaplan Meier Curves for Model Comparison in the Training Cohort***

A) Kaplan Meier survival curves based on the prognostic modeling for the 95-gene residual risk signature modeled without clinical covariates and representing patients receiving only endocrine therapy. B) Risk score estimates shown in A grouped as quartiles with each group compared against Q1. Hazard ratios were estimated using Cox proportional hazards model and significance of survival difference was estimated using the log-rank test. C) Kaplan Meier survival curves based on the prognostic modeling for the 95-gene residual risk signature modeled with clinical covariates including age, grade, pathological tumor size and nodal status; and representing patients receiving only endocrine therapy. D) Risk score estimates shown in C grouped as quartiles with each group compared against Q1. Hazard ratios were estimated using Cox proportional hazards model and significance of survival difference was estimated using the log-rank test. E) Kaplan Meier survival curves based on the prognostic modeling for the 95-gene residual risk signature modeled only with nodal status as the only clinical covariate among patients receiving only endocrine therapy. F) Risk score estimates shown in E grouped as quartiles with each group compared against Q1. Hazard ratios were estimated using Cox proportional hazards model and significance of survival difference was estimated using the log-rank test.

***Supplementary Figure 3: Kaplan Meier Curves for Model Comparison in the Validation Cohort***

A) Kaplan Meier survival curves based on the prognostic modeling for the 95-gene residual risk signature modeled without clinical covariates and representing patients receiving only endocrine therapy. B) Risk score estimates shown in A grouped as quartiles with each group compared against Q1. Hazard ratios were estimated using Cox proportional hazards model and significance of survival difference was estimated using the log-rank test. C) Kaplan Meier survival curves based on the prognostic modeling for the 95-gene residual risk signature modeled with clinical covariates including age, grade, pathological tumor size and nodal status; and representing patients receiving only endocrine therapy. D) Risk score estimates shown in C grouped as quartiles with each group compared against Q1. Hazard ratios were estimated using Cox proportional hazards model and significance of survival difference was estimated using the log-rank test.

***Supplementary Figure 4: Validation of the 95-Gene Residual Risk Signature in Chemotherapy-treated and Non-Chemotherapy-treated Patients in the Validation Cohort***

A) Kaplan Meier survival curves based on the prognostic modeling of the 95-gene residual risk signature including nodal status in the validation cohort including patients who received adjuvant chemotherapy and adjusted for chemotherapy. B) Risk score estimates shown in A grouped as quartiles with each group compared against Q1. Hazard ratios were estimated using Cox proportional hazards model and significance of survival difference was estimated using the log-rank test. C) Survival curves as shown in A and distinguishing patients identified as high- or low-risk and treatment with adjuvant chemotherapy and adjusted for chemotherapy.

***Supplementary Figure 5: Validation of the 95-Gene Residual Risk Signature in HER2-Positive and HER2-Negative Patients in the Validation Cohort.***

A) Kaplan Meier survival curves based on the prognostic modeling of the 95-gene residual risk signature including nodal status in the validation cohort of patients who did not receive adjuvant chemotherapy stratified by HER2 status. B) Kaplan Meier survival curves based on the prognostic modeling of the 95-gene residual risk signature including nodal status in the validation of cohort patients who did not receive adjuvant chemotherapy stratified by HER2-negative patients. C) Kaplan Meier survival curves based on the prognostic modeling of the 95-gene residual risk signature including nodal status in the validation cohort of patients who did not receive adjuvant chemotherapy stratified by HER2-positive patients.

***Supplementary Figure 6: Kaplan Meier Survival Analyses of Current Commercial and Academic Multiparametric Tests***

Shown in the figures are the Kaplan Meier survival curves based on the expression of genes modeled for the various multiparametric tests in the validation cohort. A) Results of the Prosigna test of patients in the validation cohort. Patients identified as low- and intermediate-risk show similar survival, with high-risk patients showing worse DRFS. B) Kaplan Meier survival of patients according to the intrinsic subtyping results based on the Prosigna multiparametric algorithm. C) Kaplan Meier survival analyses of the validation cohort based on OncotypeDx-*like* expression analyses, dichotomized using a risk score (RS) cut off of 25. D) Kaplan Meier survival analyses of the validation cohort based on MammaPrint-*like* expression analyses. E) Kaplan Meier survival analyses of the validation cohort based on Genomic Grade Index-like expression analyses. F) Kaplan Meier survival analyses of the validation cohort patients defined as low- and high-risk based on the RNA expression values of the IHC4 genes (ER, PgR, Ki67 and HER2). G) Kaplan Meier survival analyses of the validation cohort patients defined as low- and high-risk based on the protein expression values of the IHC4 genes (ER, PgR, Ki67 and HER2).

***Supplementary Figure 7. Putative Stratification of Patients to Novel Therapeutics using the 95-Gene Signature of Residual Risk***

Shown is a putative clinical trial design based on the 95-gene signature to targeted therapies identified by *in silico* pathway analyses based on our expression profiling. In this schema, patients identified as low-risk by the signature receive endocrine treatment only. Those deemed as high-risk, along with the integration of other genomic markers such as gene mutational status and copy-number, are then triaged to targeted treatment directed at the pathways driving their cancer.

***Supplementary Figure 8: TEAM Trial Schema and Patient Samples***

A) Trial schema for the Tamoxifen and Exemestane Adjuvant Multinational Trial (TEAM) pathology cohort. Eligible patients were randomized to receive either Tamoxifen for 2.5 years followed by Exemestane for the remaining 2.5 years; or Exemestane for 5 years. B) Summary of statistical power in the TEAM cohort. C) Summary of samples collected and processed for the current study.

***Supplementary Figure 9: Pre-processing Methods Rankings of Normalization Strategies***

Preprocessing TEAM cohort. Heatmap shows ranking of preprocessing methods based on their ability to maximise molecular differences between HER2+ve and HER2-ve profiles, while minimizing batch effects. For 252 combinations of preprocessing methods, two rankings were established as per above criteria, and subsequently aggregated using the rank product. The heatmap is sorted based on the aggregate rank with the most effective preprocessing parameters appearing at the top.

***Supplementary Tables***

***Supplementary Table 1: Clinical Description of Training and Validation Cohorts (Endocrine-Treated and Endocrine-Treated with Adjuvant Chemotherapy)***

|  | **Overall** | **Training Cohort** | **Validation Cohort** | **P (Training vs. Validation)** |
| --- | --- | --- | --- | --- |
| **Samples** |  |  |  |  |
|  |  |  |  |  |
| **Age** |  |  |  | 2.48 x10^-2^ |
| ≥55 | 3322 (86.8%) | 705 (89.2%) | 2617 (86.2%) |  |
| <55 | 503 (13.2%) | 85 (10.8%) | 418 (13.8%) |  |
|  |  |  |  |  |
| **Grade** |  |  |  | 1.09 x10^-3^ |
| 1 | 427 (11.7%) | 66 (8.7%) | 361 (12.5%) |  |
| 2 | 1945 (53.4%) | 444 (58.5%) | 1501 (52.0%) |  |
| 3 | 1271 (34.9%) | 249 (32.8%) | 1022 (35.4%) |  |
|  |  |  |  |  |
| **Number of positive nodes** |  |  |  | 3.92 x10^-8^ |
| 0 | 1466 (39.3%) | 375 (49.0%) | 1091 (36.8%) |  |
| 1-3 | 1662 (44.5%) | 289 (37.7%) | 1373 (46.3%) |  |
| 4-9 | 416 (11.1%) | 71 (9.3%) | 345 (11.6%) |  |
| 10+ | 190 (5.1%) | 31 (4.0%) | 159 (5.4%) |  |
|  |  |  |  |  |
| **Pathological Size (Categorical)** |  |  |  | 3.59 x10^-10^ |
| ≤2cm | 1806 (47.3%) | 448 (56.8%) | 1358 (44.8%) |  |
| >2 & ≤5cm | 1787 (46.8%) | 317 (40.2%) | 1470 (48.5%) |  |
| >5cm | 226 (5.9%) | 24 (3.0%) | 202 (6.7%) |  |
|  |  |  |  |  |
| **HER2** |  |  |  | 8.09 x10^-2^ |
| Negative | 3202 (87.0%) | 659 (85.1%) | 2543 (87.5%) |  |
| Positive | 477 (13.0%) | 115 (14.9%) | 362 (12.5%) |  |

***Supplementary Table 2 : Univariate Results of Prognostically Significant Genes in the 95-Gene Signature of the Validation Cohort***

| **Validation: Endocrine-treated** | | | | | | **Validation: Endocrine-treated + Adjuvant Chemotherapy** | | | | | |
| --- | --- | --- | --- | --- | --- | --- | --- | --- | --- | --- | --- |
|  | **Coef** | **HR** | **HR.95L** | **HR.95U** | **P** |  | **Coef** | **HR** | **HR.95L** | **HR.95U** | **P** |
| **ACTR3B** | -0.1555 | 0.856 | 0.669 | 1.096 | 2.19 x 10^-1^ | **ACTR3B** | -0.2033 | 0.816 | 0.675 | 0.986 | 3.50 x 10^-2^ |
| **ANLN** | 1.1135 | 3.045 | 2.302 | 4.027 | 5.99 x 10^-15^ | **ANLN** | 0.9639 | 2.622 | 2.131 | 3.228 | 9.11 x 10^-20^ |
| **ASPM** | 0.7400 | 2.096 | 1.615 | 2.721 | 2.71 x 10^-8^ | **ASPM** | 0.7743 | 2.169 | 1.774 | 2.653 | 4.66 x 10^-14^ |
| **AURKA** | 0.9042 | 2.47 | 1.886 | 3.236 | 5.10 x 10^-11^ | **AURKA** | 0.6931 | 2 | 1.638 | 2.44 | 9.19 x 10^-12^ |
| **BAG1** | -0.1416 | 0.868 | 0.678 | 1.111 | 2.60 x 10^-1^ | **BAG1** | -0.1031 | 0.902 | 0.746 | 1.089 | 2.83 x 10^-1^ |
| **BCL2** | -0.7423 | 0.476 | 0.367 | 0.618 | 2.54 x 10^-8^ | **BCL2** | -0.7508 | 0.472 | 0.386 | 0.576 | 1.90 x 10^-13^ |
| **BIRC5** | 1.0784 | 2.94 | 2.227 | 3.883 | 2.91 x 10^-14^ | **BIRC5** | 0.9666 | 2.629 | 2.135 | 3.238 | 8.70 x 10^-20^ |
| **BUB1B** | 1.1869 | 3.277 | 2.464 | 4.358 | 3.30 x 10^-16^ | **BUB1B** | 0.9888 | 2.688 | 2.181 | 3.313 | 1.89 x 10^-20^ |
| **CCNB1** | 0.9670 | 2.63 | 2.004 | 3.452 | 3.20 x 10^-12^ | **CCNB1** | 0.9103 | 2.485 | 2.023 | 3.052 | 4.31 x 10^-18^ |
| **CCNB2** | 0.8658 | 2.377 | 1.818 | 3.106 | 2.38 x 10^-10^ | **CCNB2** | 0.8817 | 2.415 | 1.967 | 2.965 | 3.87 x 10^-17^ |
| **CCND1** | 0.0602 | 1.062 | 0.83 | 1.36 | 6.31 x 10^-1^ | **CCND1** | 0.0862 | 1.09 | 0.902 | 1.316 | 3.74 x 10^-1^ |
| **CCNE1** | 0.7041 | 2.022 | 1.559 | 2.623 | 1.13 x 10^-7^ | **CCNE1** | 0.7376 | 2.091 | 1.711 | 2.555 | 5.69 x 10^-13^ |
| **CCNE2** | 0.6941 | 2.002 | 1.544 | 2.595 | 1.58 x 10^-7^ | **CCNE2** | 0.6851 | 1.984 | 1.627 | 2.42 | 1.34 x 10^-11^ |
| **CDC20** | 0.9099 | 2.484 | 1.897 | 3.254 | 3.89 x 10^-11^ | **CDC20** | 0.8616 | 2.367 | 1.929 | 2.904 | 1.45 x 10^-16^ |
| **CDC6** | 0.8684 | 2.383 | 1.825 | 3.112 | 1.81 x 10^-10^ | **CDC6** | 0.7056 | 2.025 | 1.66 | 2.472 | 3.80 x 10^-12^ |
| **CDCA7** | 0.5481 | 1.73 | 1.342 | 2.23 | 2.35 x 10^-5^ | **CDCA7** | 0.4440 | 1.559 | 1.286 | 1.89 | 6.39 x 10^-6^ |
| **CDH3** | 0.1748 | 1.191 | 0.93 | 1.525 | 1.67 x 10^-1^ | **CDH3** | 0.0535 | 1.055 | 0.873 | 1.274 | 5.79 x 10^-1^ |
| **CDK1** | 0.9768 | 2.656 | 2.023 | 3.487 | 1.97 x 10^-12^ | **CDK1** | 0.8842 | 2.421 | 1.974 | 2.971 | 2.31 x 10^-17^ |
| **CENPA** | 0.9817 | 2.669 | 2.033 | 3.503 | 1.54 x 10^-12^ | **CENPA** | 0.8312 | 2.296 | 1.874 | 2.812 | 9.56 x 10^-16^ |
| **CENPF** | 1.0178 | 2.767 | 2.105 | 3.636 | 2.89 x 10^-13^ | **CENPF** | 0.9075 | 2.478 | 2.018 | 3.043 | 4.54 x 10^-18^ |
| **CEP55** | 1.1762 | 3.242 | 2.442 | 4.306 | 4.35 x 10^-16^ | **CEP55** | 1.0396 | 2.828 | 2.291 | 3.491 | 4.08 x 10^-22^ |
| **CMC2** | 0.4916 | 1.635 | 1.268 | 2.108 | 1.48 x 10^-4^ | **CMC2** | 0.5642 | 1.758 | 1.445 | 2.138 | 1.68 x 10^-8^ |
| **CX3CR1** | -0.7236 | 0.485 | 0.374 | 0.629 | 4.70 x 10^-8^ | **CX3CR1** | -0.5745 | 0.563 | 0.463 | 0.684 | 7.73 x 10^-9^ |
| **CXXC5** | 0.5562 | 1.744 | 1.353 | 2.249 | 1.80 x 10^-5^ | **CXXC5** | 0.3723 | 1.451 | 1.198 | 1.758 | 1.40 x 10^-4^ |
| **DHX58** | -0.2244 | 0.799 | 0.624 | 1.024 | 7.66 x 10^-2^ | **DHX58** | -0.2319 | 0.793 | 0.656 | 0.959 | 1.67 x 10^-2^ |
| **DIAPH3** | 0.5271 | 1.694 | 1.314 | 2.183 | 4.79 x 10^-5^ | **DIAPH3** | 0.4612 | 1.586 | 1.307 | 1.924 | 2.88 x 10^-6^ |
| **DTL** | 0.6286 | 1.875 | 1.45 | 2.426 | 1.70 x 10^-6^ | **DTL** | 0.6334 | 1.884 | 1.547 | 2.295 | 3.15 x 10^-10^ |
| **EBF4** | 0.1756 | 1.192 | 0.931 | 1.526 | 1.64 x 10^-1^ | **EBF4** | 0.0602 | 1.062 | 0.879 | 1.283 | 5.32 x 10^-1^ |
| **ECT2** | 1.1072 | 3.026 | 2.289 | 4.002 | 7.87 x 10^-15^ | **ECT2** | 1.0392 | 2.827 | 2.288 | 3.491 | 5.32 x 10^-22^ |
| **EGFR** | -0.0866 | 0.917 | 0.717 | 1.174 | 4.92 x 10^-1^ | **EGFR** | -0.0790 | 0.924 | 0.765 | 1.117 | 4.14 x 10^-1^ |
| **EGLN1** | 0.2769 | 1.319 | 1.029 | 1.692 | 2.90 x 10^-2^ | **EGLN1** | 0.2562 | 1.292 | 1.068 | 1.562 | 8.36 x 10^-3^ |
| **ERBB3** | -0.2256 | 0.798 | 0.623 | 1.022 | 7.32 x 10^-2^ | **ERBB3** | -0.1948 | 0.823 | 0.681 | 0.994 | 4.35 x 10^-2^ |
| **ERBB4** | -0.2095 | 0.811 | 0.633 | 1.039 | 9.72 x 10^-2^ | **ERBB4** | -0.1649 | 0.848 | 0.702 | 1.025 | 8.76 x 10^-2^ |
| **ESM1** | 0.4781 | 1.613 | 1.253 | 2.077 | 2.06 x 10^-4^ | **ESM1** | 0.4600 | 1.584 | 1.306 | 1.921 | 2.91 x 10^-6^ |
| **ESPL1** | 0.8198 | 2.27 | 1.743 | 2.957 | 1.22 x 10^-9^ | **ESPL1** | 0.7090 | 2.032 | 1.665 | 2.48 | 3.03 x 10^-12^ |
| **EXO1** | 0.9435 | 2.569 | 1.96 | 3.369 | 8.66 x 10^-12^ | **EXO1** | 0.9066 | 2.476 | 2.015 | 3.043 | 6.70 x 10^-18^ |
| **FGF18** | -0.1590 | 0.853 | 0.666 | 1.092 | 2.08 x 10^-1^ | **FGF18** | -0.1744 | 0.84 | 0.695 | 1.016 | 7.22 x 10^-2^ |
| **FOXC1** | 0.0109 | 1.011 | 0.79 | 1.293 | 9.32 x 10^-1^ | **FOXC1** | 0.0305 | 1.031 | 0.853 | 1.245 | 7.54 x 10^-1^ |
| **FRY** | -0.6444 | 0.525 | 0.406 | 0.678 | 8.84 x 10^-7^ | **FRY** | -0.4829 | 0.617 | 0.508 | 0.749 | 9.96 x 10^-7^ |
| **GMPS** | 0.4492 | 1.567 | 1.218 | 2.016 | 4.77 x 10^-4^ | **GMPS** | 0.4035 | 1.497 | 1.235 | 1.814 | 3.93 x 10^-5^ |
| **GNAZ** | 0.7222 | 2.059 | 1.588 | 2.669 | 4.91 x 10^-8^ | **GNAZ** | 0.5755 | 1.778 | 1.463 | 2.162 | 7.48 x 10^-9^ |
| **GSK3B** | 0.2919 | 1.339 | 1.044 | 1.716 | 2.15 x 10^-2^ | **GSK3B** | 0.2814 | 1.325 | 1.095 | 1.603 | 3.76 x 10^-3^ |
| **GSTM3** | -0.5888 | 0.555 | 0.43 | 0.717 | 6.74 x 10^-6^ | **GSTM3** | -0.4797 | 0.619 | 0.51 | 0.751 | 1.19 x 10^-6^ |
| **JHDM1D** | -0.0523 | 0.949 | 0.741 | 1.214 | 6.75 x 10^-1^ | **JHDM1D** | -0.0111 | 0.989 | 0.818 | 1.194 | 9.05 x 10^-1^ |
| **KIF2C** | 0.9620 | 2.617 | 1.994 | 3.435 | 4.16 x 10^-12^ | **KIF2C** | 0.8078 | 2.243 | 1.832 | 2.746 | 5.13 x 10^-15^ |
| **KPNA2** | 0.7766 | 2.174 | 1.671 | 2.829 | 7.46 x 10^-9^ | **KPNA2** | 0.6790 | 1.972 | 1.617 | 2.404 | 1.90 x 10^-11^ |
| **KRT14** | -0.4292 | 0.651 | 0.506 | 0.836 | 7.99 x 10^-4^ | **KRT14** | -0.3230 | 0.724 | 0.598 | 0.876 | 8.91 x 10^-4^ |
| **KRT8** | 0.4756 | 1.609 | 1.251 | 2.07 | 2.11 x 10^-4^ | **KRT8** | 0.3148 | 1.37 | 1.132 | 1.657 | 1.20 x 10^-3^ |
| **LETMD1** | -0.2744 | 0.76 | 0.593 | 0.974 | 3.01 x 10^-2^ | **LETMD1** | -0.1696 | 0.844 | 0.699 | 1.02 | 7.96 x 10^-2^ |
| **LIN9** | 0.3407 | 1.406 | 1.095 | 1.805 | 7.52 x 10^-3^ | **LIN9** | 0.4266 | 1.532 | 1.263 | 1.857 | 1.48 x 10^-5^ |
| **LPCAT1** | 0.4285 | 1.535 | 1.194 | 1.974 | 8.25 x 10^-4^ | **LPCAT1** | 0.3974 | 1.488 | 1.228 | 1.803 | 4.95 x 10^-5^ |
| **MAD2L1** | 0.6714 | 1.957 | 1.51 | 2.537 | 3.93 x 10^-7^ | **MAD2L1** | 0.5800 | 1.786 | 1.468 | 2.174 | 7.17 x 10^-9^ |
| **MAPT** | -0.8119 | 0.444 | 0.341 | 0.578 | 1.64 x 10^-9^ | **MAPT** | -0.7700 | 0.463 | 0.379 | 0.566 | 5.53 x 10^-14^ |
| **MCM10** | 1.0946 | 2.988 | 2.263 | 3.947 | 1.23 x 10^-14^ | **MCM10** | 1.0163 | 2.763 | 2.24 | 3.407 | 2.09 x 10^-21^ |
| **MCM2** | 0.8290 | 2.291 | 1.757 | 2.987 | 9.05 x 10^-10^ | **MCM2** | 0.7495 | 2.116 | 1.732 | 2.586 | 2.39 x 10^-13^ |
| **MCM6** | 0.8320 | 2.298 | 1.763 | 2.995 | 7.71 x 10^-10^ | **MCM6** | 0.7710 | 2.162 | 1.769 | 2.643 | 5.37 x 10^-14^ |
| **MDM2** | -0.2497 | 0.779 | 0.608 | 0.998 | 4.78 x 10^-2^ | **MDM2** | -0.2971 | 0.743 | 0.614 | 0.898 | 2.19 x 10^-3^ |
| **MELK** | 0.8858 | 2.425 | 1.855 | 3.17 | 9.22 x 10^-11^ | **MELK** | 0.7505 | 2.118 | 1.733 | 2.588 | 2.23 x 10^-13^ |
| **MKI67** | 1.1049 | 3.019 | 2.283 | 3.993 | 9.27 x 10^-15^ | **MKI67** | 1.0310 | 2.804 | 2.272 | 3.462 | 8.68 x 10^-22^ |
| **MMP11** | 0.3556 | 1.427 | 1.112 | 1.831 | 5.23 x 10^-3^ | **MMP11** | 0.3988 | 1.49 | 1.23 | 1.805 | 4.51 x 10^-5^ |
| **MMP9** | 0.4662 | 1.594 | 1.239 | 2.051 | 2.91 x 10^-4^ | **MMP9** | 0.3457 | 1.413 | 1.167 | 1.711 | 3.89 x 10^-4^ |
| **MS4A7** | -0.5834 | 0.558 | 0.432 | 0.721 | 7.74 x 10^-6^ | **MS4A7** | -0.5192 | 0.595 | 0.49 | 0.723 | 1.67 x 10^-7^ |
| **MYBL2** | 1.1356 | 3.113 | 2.347 | 4.128 | 3.17 x 10^-15^ | **MYBL2** | 0.9616 | 2.616 | 2.124 | 3.221 | 1.36 x 10^-19^ |
| **NAT1** | -0.6773 | 0.508 | 0.392 | 0.658 | 2.82 x 10^-7^ | **NAT1** | -0.5551 | 0.574 | 0.472 | 0.698 | 2.47 x 10^-8^ |
| **NDC80** | 0.7314 | 2.078 | 1.601 | 2.697 | 3.96 x 10^-8^ | **NDC80** | 0.6811 | 1.976 | 1.62 | 2.41 | 1.76 x 10^-11^ |
| **NEK2** | 0.8734 | 2.395 | 1.834 | 3.128 | 1.42 x 10^-10^ | **NEK2** | 0.8224 | 2.276 | 1.858 | 2.788 | 1.88 x 10^-15^ |
| **NUF2** | 0.4996 | 1.648 | 1.279 | 2.123 | 1.10 x 10^-4^ | **NUF2** | 0.5527 | 1.738 | 1.43 | 2.113 | 2.84 x 10^-8^ |
| **NUSAP1** | 1.0842 | 2.957 | 2.236 | 3.91 | 2.80 x 10^-14^ | **NUSAP1** | 1.0210 | 2.776 | 2.249 | 3.427 | 2.10 x 10^-21^ |
| **ORC6** | 0.9851 | 2.678 | 2.038 | 3.519 | 1.59 x 10^-12^ | **ORC6** | 0.8489 | 2.337 | 1.906 | 2.865 | 3.12 x 10^-16^ |
| **PGR** | -0.9039 | 0.405 | 0.31 | 0.53 | 3.93 x 10^-11^ | **PGR** | -0.9571 | 0.384 | 0.312 | 0.473 | 1.48 x 10^-19^ |
| **PHGDH** | 0.3053 | 1.357 | 1.058 | 1.741 | 1.63 x 10^-2^ | **PHGDH** | 0.3866 | 1.472 | 1.215 | 1.783 | 7.95 x 10^-5^ |
| **PITRM1** | -0.0555 | 0.946 | 0.739 | 1.21 | 6.57 x 10^-1^ | **PITRM1** | -0.0640 | 0.938 | 0.777 | 1.134 | 5.10 x 10^-1^ |
| **PLK1** | 0.7830 | 2.188 | 1.682 | 2.848 | 5.62 x 10^-9^ | **PLK1** | 0.7115 | 2.037 | 1.668 | 2.486 | 2.78 x 10^-12^ |
| **PRC1** | 0.8875 | 2.429 | 1.858 | 3.175 | 8.46 x 10^-11^ | **PRC1** | 0.7880 | 2.199 | 1.797 | 2.691 | 2.10 x 10^-14^ |
| **PTTG1** | 0.9936 | 2.701 | 2.055 | 3.549 | 1.03 x 10^-12^ | **PTTG1** | 0.9030 | 2.467 | 2.008 | 3.03 | 7.88 x 10^-18^ |
| **QSOX2** | 0.3988 | 1.49 | 1.159 | 1.915 | 1.84 x 10^-3^ | **QSOX2** | 0.3674 | 1.444 | 1.192 | 1.749 | 1.72 x 10^-4^ |
| **RACGAP1** | 0.6339 | 1.885 | 1.457 | 2.439 | 1.39 x 10^-6^ | **RACGAP1** | 0.4867 | 1.627 | 1.341 | 1.975 | 8.32 x 10^-7^ |
| **RFC4** | 0.5435 | 1.722 | 1.335 | 2.221 | 2.88 x 10^-5^ | **RFC4** | 0.5110 | 1.667 | 1.373 | 2.025 | 2.49 x 10^-7^ |
| **RRM2** | 1.0699 | 2.915 | 2.207 | 3.849 | 4.61 x 10^-14^ | **RRM2** | 0.8725 | 2.393 | 1.951 | 2.936 | 5.90 x 10^-17^ |
| **RUNDC1** | -0.6311 | 0.532 | 0.411 | 0.687 | 1.36 x 10^-6^ | **RUNDC1** | -0.5586 | 0.572 | 0.471 | 0.695 | 1.96 x 10^-8^ |
| **SCUBE2** | -0.5745 | 0.563 | 0.436 | 0.727 | 1.02 x 10^-5^ | **SCUBE2** | -0.5092 | 0.601 | 0.495 | 0.73 | 2.76 x 10^-7^ |
| **SERF1A** | -0.0030 | 0.997 | 0.779 | 1.276 | 9.83 x 10^-1^ | **SERF1A** | -0.0161 | 0.984 | 0.815 | 1.188 | 8.66 x 10^-1^ |
| **SFRP1** | -0.3682 | 0.692 | 0.539 | 0.889 | 3.92 x 10^-3^ | **SFRP1** | -0.2904 | 0.748 | 0.618 | 0.905 | 2.81 x 10^-3^ |
| **SLC7A5** | 0.7381 | 2.092 | 1.61 | 2.718 | 3.23 x 10^-8^ | **SLC7A5** | 0.6119 | 1.844 | 1.515 | 2.245 | 1.08 x 10^-9^ |
| **SPEF1** | -0.3038 | 0.738 | 0.576 | 0.947 | 1.68 x 10^-2^ | **SPEF1** | -0.3383 | 0.713 | 0.589 | 0.863 | 5.23 x 10^-4^ |
| **STK32B** | -0.3481 | 0.706 | 0.55 | 0.906 | 6.24 x 10^-3^ | **STK32B** | -0.3453 | 0.708 | 0.585 | 0.857 | 4.03 x 10^-4^ |
| **STMN1** | 0.8842 | 2.421 | 1.854 | 3.162 | 8.38 x 10^-11^ | **STMN1** | 0.7328 | 2.081 | 1.704 | 2.54 | 6.09 x 10^-13^ |
| **TGFB3** | -0.3439 | 0.709 | 0.552 | 0.91 | 6.88 x 10^-3^ | **TGFB3** | -0.2666 | 0.766 | 0.634 | 0.927 | 6.12 x 10^-3^ |
| **TP53** | -0.3552 | 0.701 | 0.546 | 0.9 | 5.35 x 10^-3^ | **TP53** | -0.3052 | 0.737 | 0.609 | 0.892 | 1.74 x 10^-3^ |
| **TRMT2A** | -0.2758 | 0.759 | 0.592 | 0.974 | 3.00 x 10^-2^ | **TRMT2A** | -0.1863 | 0.83 | 0.687 | 1.004 | 5.47 x 10^-2^ |
| **TYMS** | 0.6560 | 1.927 | 1.489 | 2.495 | 6.31 x 10^-7^ | **TYMS** | 0.6021 | 1.826 | 1.501 | 2.222 | 1.81 x 10^-9^ |
| **UBE2C** | 0.6429 | 1.902 | 1.47 | 2.461 | 9.88 x 10^-7^ | **UBE2C** | 0.6785 | 1.971 | 1.617 | 2.404 | 1.95 x 10^-11^ |
| **UBE2T** | 1.0753 | 2.931 | 2.22 | 3.871 | 3.41 x 10^-14^ | **UBE2T** | 0.9532 | 2.594 | 2.108 | 3.192 | 2.29 x 10^-19^ |
| **WISP1** | -0.2850 | 0.752 | 0.587 | 0.965 | 2.48 x 10^-2^ | **WISP1** | -0.2107 | 0.81 | 0.67 | 0.979 | 2.94 x 10^-2^ |
| **ZNF385B** | -0.2536 | 0.776 | 0.605 | 0.994 | 4.50 x 10^-2^ | **ZNF385B** | -0.1948 | 0.823 | 0.681 | 0.995 | 4.38 x 10^-2^ |

***Supplementary Table 3: Summary of Risk Scores Across Different Tests of the Validation Cohort***

|  | **95-Gene Signature** | **MammaPrint-*like*** | **OncotypeDx-*like***  **(RS cut-off 25)** | **Prosigna-*like*** | **Genomic Grade Index-*like*** | **mRNA-IHC4** |
| --- | --- | --- | --- | --- | --- | --- |
| **Low Risk** | n=822 | n=1125 | n=936 | n=194 | n=955 | n=569 |
| **Intermediate Risk** | NA | NA | NA | n=744 | NA | NA |
| **High Risk** | n=1102 | n=848 | n=1037 | n=1033 | n=1018 | n=1404 |
| **Luminal A** | NA | NA | NA | n=777 | NA | NA |
| **Luminal B** | NA | NA | NA | n=502 | NA | NA |
| **Basal-like** | NA | NA | NA | n=352 | NA | NA |
| **HER2 enriched-like** | NA | NA | NA | n=342 | NA | NA |

***Supplementary Table 4: Multiparametric Test Concordance in the Validation Cohort***

|  | | **IHC4 Protein** | | **Genomic Grade Index-*like*** | | **MammaPrint-*like*** | | **Prosigna-*like*** | | **Oncotype DX-*like*** | |
| --- | --- | --- | --- | --- | --- | --- | --- | --- | --- | --- | --- |
|  |  | **Low** | **High** | **Low** | **High** | **Low** | **High** | **Low** | **High** | **Low** | **High** |
| **Genomic Grade Index-*like*** | **Low** | 652 | 234 |  |  |  |  |  |  |  |  |
|  | **High** | 479 | 490 |  |  |  |  |  |  |  |  |
| **MammaPrint-*like*** | **Low** | 762 | 291 | 838 | 287 |  |  |  |  |  |  |
|  | **High** | 369 | 433 | 117 | 731 |  |  |  |  |  |  |
| **Prosigna-*like*** | **Low** | 647 | 221 | 808 | 130 | 844 | 94 |  |  |  |  |
|  | **High** | 483 | 502 | 146 | 887 | 280 | 753 |  |  |  |  |
| **Oncotype DX-*like*** | **Low** | 719 | 160 | 661 | 275 | 758 | 178 | 659 | 277 |  |  |
|  | **High** | 412 | 564 | 294 | 743 | 367 | 670 | 279 | 756 |  |  |
| **95-Gene Signature** | **Low** | 585 | 176 | 687 | 135 | 746 | 76 | 705 | 116 | 599 | 223 |
|  | **High** | 517 | 533 | 241 | 861 | 346 | 756 | 207 | 894 | 312 | 790 |

***Supplementary Table 5: Normalized RNA Abundance Values per Gene Within Pathway Modules showing Relative RNA Abundance in the Validation Cohort***

| **Module 1** | | | | | | | | | | | |
| --- | --- | --- | --- | --- | --- | --- | --- | --- | --- | --- | --- |
| **Gene** | **Low** | **High** | **FC** | **P** | **Q** | **Gene** | **Low** | **High** | **FC** | **P** | **Q** |
| **BIRC5** | 5.63 | 7.07 | 1.44 | 7.23 x 10^-180^ | 1.37 x 10^-178^ | **ESPL1** | 4.15 | 5.38 | 1.23 | 2.38 x 10^-167^ | 2.06 x 10^-166^ |
| **BUB1B** | 4.60 | 5.74 | 1.15 | 1.78 x 10^-180^ | 4.24 x 10^-179^ | **KIF2C** | 3.94 | 5.01 | 1.06 | 1.80 x 10^-139^ | 6.84 x 10^-139^ |
| **CCNB1** | 6.25 | 7.30 | 1.05 | 1.76 x 10^-173^ | 2.39 x 10^-172^ | **MAD2L1** | 4.92 | 5.58 | 0.66 | 1.12 x 10^-107^ | 2.95 x 10^-107^ |
| **CCNB2** | 4.34 | 5.45 | 1.11 | 6.28 x 10^-153^ | 2.84 x 10^-152^ | **NDC80** | 3.64 | 4.65 | 1.01 | 6.61 x 10^-137^ | 2.42 x 10^-136^ |
| **CDC20** | 5.48 | 6.48 | 1.00 | 1.68 x 10^-153^ | 8.00 x 10^-153^ | **NUF2** | 3.59 | 4.37 | 0.78 | 1.70 x 10^-83^ | 3.85 x 10^-83^ |
| **CENPA** | 3.87 | 5.02 | 1.15 | 1.47 x 10^-149^ | 6.34 x 10^-149^ | **PTTG1** | 6.25 | 7.24 | 0.99 | 9.36 x 10^-175^ | 1.48 x 10^-173^ |
| **CENPF** | 6.65 | 7.80 | 1.15 | 4.51 x 10^-167^ | 3.57 x 10^-166^ | **STMN1** | 7.55 | 8.24 | 0.70 | 7.53 x 10^-103^ | 1.93 x 10^-102^ |
| **Module 2** | | | | | | | | | | | |
| **Gene** | **Low** | **High** | **FC** | **P** | **Q** | **Gene** | **Low** | **High** | **FC** | **P** | **Q** |
| **BAG1** | 6.01 | 5.96 | 0.04 | 6.01 x 10^-2^ | 6.80 x 10^-2^ | **GSK3B** | 7.73 | 7.82 | 0.10 | 4.22 x 10^-10^ | 5.20 x 10^-10^ |
| **BCL2** | 7.06 | 6.67 | 0.39 | 1.60 x 10^-22^ | 2.17 x 10^-22^ | **MAPT** | 8.24 | 7.50 | 0.74 | 4.38 x 10^-34^ | 7.43 x 10^-34^ |
| **CCNE1** | 4.27 | 4.96 | 0.69 | 7.17 x 10^-91^ | 1.70 x 10^-90^ | **MDM2** | 8.39 | 8.42 | 0.03 | 4.86 x 10^-1^ | 5.13 x 10^-1^ |
| **EGFR** | 5.79 | 5.20 | 0.59 | 2.28 x 10^-29^ | 3.61 x 10^-29^ | **RRM2** | 6.14 | 7.35 | 1.22 | 2.66 x 10^-164^ | 1.94 x 10^-163^ |
| **ERBB3** | 7.97 | 7.99 | 0.02 | 7.16 x 10^-1^ | 7.31 x 10^-1^ | **TP53** | 7.06 | 6.94 | 0.12 | 1.12 x 10^-7^ | 1.35 x 10^-7^ |
| **ERBB4** | 5.93 | 5.46 | 0.48 | 2.17 x 10^-17^ | 2.86 x 10^-17^ | **TYMS** | 6.83 | 7.67 | 0.84 | 8.16 x 10^-131^ | 2.50 x 10^-130^ |
| **FGF18** | 4.52 | 3.99 | 0.53 | 5.15 x 10^-25^ | 7.53 x 10^-25^ |  |  |  |  |  |  |
| **Module 3** | | | | | | | | | | | |
| **Gene** | **Low** | **High** | **FC** | **P** | **Q** | **Gene** | **Low** | **High** | **FC** | **P** | **Q** |
| **ASPM** | 4.35 | 5.49 | 1.15 | 2.59 x 10^-155^ | 1.29 x 10^-154^ | **NEK2** | 5.89 | 7.11 | 1.21 | 7.06 x 10^-169^ | 6.71 x 10^-168^ |
| **AURKA** | 5.09 | 5.76 | 0.68 | 8.61 x 10^-115^ | 2.34 x 10^-114^ | **PLK1** | 4.78 | 5.97 | 1.19 | 5.68 x 10^-156^ | 3.18 x 10^-155^ |
| **CCNE2** | 4.32 | 5.35 | 1.03 | 1.75 x 10^-115^ | 4.89 x 10^-115^ | **PRC1** | 5.23 | 6.24 | 1.01 | 1.67 x 10^-155^ | 8.82 x 10^-155^ |
| **CDK1** | 5.11 | 6.26 | 1.15 | 2.52 x 10^-157^ | 1.50 x 10^-156^ | **RACGAP1** | 3.23 | 3.78 | 0.55 | 6.58 x 10^-46^ | 1.28 x 10^-45^ |
| **CEP55** | 5.03 | 6.23 | 1.20 | 2.43 x 10^-169^ | 2.88 x 10^-168^ | **UBE2C** | 7.38 | 8.10 | 0.72 | 2.79 x 10^-100^ | 6.98 x 10^-100^ |
| **ECT2** | 6.84 | 7.51 | 0.66 | 7.19 x 10^-136^ | 2.44 x 10^-135^ |  |  |  |  |  |  |
| **Module 4** | | | | | | | | | | | |
| **Gene** | **Low** | **High** | **FC** | **P** | **Q** | **Gene** | **Low** | **High** | **FC** | **P** | **Q** |
| **CCND1** | 10.63 | 10.86 | 0.23 | 1.55 x 10^-6^ | 1.82 x 10^-6^ | **MCM6** | 6.56 | 7.00 | 0.44 | 3.02 x 10^-90^ | 7.00 x 10^-90^ |
| **CDC6** | 4.65 | 5.66 | 1.01 | 2.07 x 10^-131^ | 6.56 x 10^-131^ | **MYBL2** | 5.22 | 6.94 | 1.71 | 7.49 x 10^-182^ | 2.37 x 10^-180^ |
| **LIN9** | 5.42 | 5.81 | 0.38 | 3.91 x 10^-44^ | 7.14 x 10^-44^ | **ORC6** | 3.64 | 4.72 | 1.08 | 7.86 x 10^-122^ | 2.33 x 10^-121^ |
| **MCM10** | 4.13 | 5.30 | 1.17 | 9.54 x 10^-164^ | 6.47 x 10^-163^ | **RFC4** | 5.73 | 6.20 | 0.47 | 7.71 x 10^-72^ | 1.70 x 10^-71^ |
| **MCM2** | 5.26 | 5.98 | 0.72 | 4.90 x 10^-119^ | 1.41 x 10^-118^ | **UBE2T** | 5.67 | 6.77 | 1.10 | 6.07 x 10^-169^ | 6.41 x 10^-168^ |
| **Module 5** | | | | | | | | | | | |
| **Gene** | **Low** | **High** | **FC** | **P** | **Q** | **Gene** | **Low** | **High** | **FC** | **P** | **Q** |
| **CDH3** | 5.12 | 5.09 | 0.03 | 4.99 x 10^-1^ | 5.21 x 10^-1^ | **MMP9** | 6.73 | 7.56 | 0.84 | 1.97 x 10^-26^ | 3.06 x 10^-26^ |
| **MMP9** | 6.73 | 7.56 | 0.84 | 1.97 x 10^-26^ | 3.06 x 10^-26^ |  |  |  |  |  |  |
| **Module 6** | | | | | | | | | | | |
|  | **Low** | **High** | **FC** | **P** | **Q** | **Gene** | **Low** | **High** | **FC** | **P** | **Q** |
| **KPNA2** | 6.61 | 7.44 | 0.83 | 6.72 x 10^-133^ | 2.20 x 10^-132^ | **KRT8** | 10.81 | 11.13 | 0.32 | 2.48 x 10^-16^ | 3.19 x 10^-16^ |

***Supplementary Table 6. Summary of Late-Phase Development Compounds to Genes and Pathways Identified in the 95-Gene Signature of Residual Risk.***

| Gene Target | Drug/Compound Name and Organization | Phase Development | Mode of Action | Treatment conditions |
| --- | --- | --- | --- | --- |
| BCL2 | Flupirtine maleate  Lindopharm  AWD Pharma  Meda Synthetic Biologics  Bayer | Launched-1986 | Non-Opioid Analgesics  Creutzfeldt- Jakob Disease  Treatment of Multiple Sclerosis | Signal Transduction Modulators  Voltage-Gated K(V) 7 (KCNQ) Channel Activators NMDA Antagonists |
|  | Oblimersen sodium  Genta  National Cancer Institute  Merck & Co. | Pre-Registered | BCL2 Expression Inhibitors  Apoptosis Inducers | Small Cell Lung Cancer  Prostate Cancer  Lymphocytic Leukemia  Multiple Myeloma  Non-Small Cell Lung Cancer  Leukemia  Gastric Cancer  Melanoma Skin Cancer  Breast Cancer  Pancreatic Cancer  Renal Cancer  Myeloid Leukemia  Colorectal Cancer  Liver Cancer  Non- Hodgkin's Lymphoma  Solid Tumor |
|  | Venetoclax  AbbVie  Genentech | Pre-Registered | Bcl-2 Inhibitors Signal  Transduction Modulators  Apoptosis Inducers | Lymphocytic Leukemia  Multiple Myeloma  Myeloid Leukemia  Systemic Lupus Erythematosus  Agents for Non-Hodgkin's  Lymphoma |
|  | Obatoclax mesylate  National Cancer Institute  Teva | Phase III | Bcl-2 Inhibitors  Bcl-xl Inhibitors Signal  Transduction  Modulators Bcl-2-Related Protein A1 (BFL-1;BCL2A1) Inhibitors  Apoptosis Inducers | Small Cell Lung Cancer  Lymphocyti Leukemia  Multiple Myeloma  Myelodysplastic Syndrome  Non-Small Cell Lung Cancer  Lymphoma  Myeloid  Leukemia  Solid Tumors  Hematologic Agents |
|  | Alvocidib Hydrochloride  National Cancer Institute  Sanofi  Memorial Sloan- Kettering Cancer Center  Tolero Pharmaceuticals  Mayo Clinic | Phase II | Mcl-1 Inhibitors  Bcl-2 Inhibitors  CDK1 Inhibitors  Signal Transduction Modulators  CDK4 Inhibitors  CDK9/Cyclin T1 Inhibitors  CDK2 Inhibitors  CDK7 Inhibitors  Apoptosis Inducers  CDK6 Inhibitors  Survivin Inhibitors  X-Chromosome- Linked Inhibitor of Apoptosis  Protein (XIAP) Inhibitors | Prostate Cancer  Lymphocytic Leukemia  Multiple Myeloma  Sarcoma  Lung Cancer  Leukemia  Gastric Cancer  Melanoma  Breast Cancer  Ovarian Cancer  Cancer of Unspecified Body Location/System  Pancreatic Cancer  Colorectal Cancer  Renal Cancer  Myeloid Leukemia  Hematological Cancer  Liver Cancer  Non-Hodgkin's Lymphoma  Solid Tumors  Head and Neck Cancer |
|  | Bardoxolone methyl  Dartmouth College  Abbott  M.D. Anderson Cancer Center  Kyowa Hakko Kirin  Reata Pharmaceuticals | Phase II | Bcl-2 Inhibitors Nuclear Factor  Erythroid 2-Related Factor 2 (NFE2- Related Factor  2; NFE2L2; NRF2) Activators  NF-kappaB (NFKB) Activation Inhibitors  Signal Transduction Modulators  IKK-1 (IKKalpha) Inhibitors  Anti-inflammatory Drugs  Heme Oxygenase Activators  Glutathione Reductase (NADPH) Activators  Apoptosis Inducers  PPARgamma Agonists  Angiogenesis Inhibitors  Nitric Oxide (NO) Production  Inhibitors | Interstitial Lung Diseases,  Renal Diseases Inflammatory  Bowel Disease,  Melanoma  Hypertension,  Pancreatic Cancer  Rheumatoid Arthritis,  Autoimmune Diseases  Solid Tumors |
|  | (-)-Gossypol  National Cancer Institute  University of Iowa  Ohio State University  National Institutes of  Health University of Michigan  Ascentage Pharma  Ascenta | Phase II | Mcl-1 Inhibitors  Bcl-xl Inhibitors  Bcl-2 Inhibitors  Signal Transduction Modulators  Lipid Peroxidation Inhibitors  Growth Factor Modulators  Bcl-2-Related Protein A1 (BFL-1; BCL2A1) Inhibitors  Bcl-w Inhibitors  Apoptosis Inducers  11beta-Hydroxysteroid Dehydrogenase (11beta-HSD)  Inhibitors  RNA-Binding Protein  Musashi Homolog 1 (MSI1)  Inhibitors | Small Cell Lung Cancer  Prostate Cancer  Lymphocytic Leukemia  Non-Small Cell Lung  Cancer  Oncolytic Drugs  Chemopreventive Agents  Digestive/Gastrointestinal  Cancer  Antipsoriatics  Glioblastoma Multiforme  Non-Hodgkin's  Lymphoma  Head and Neck Cancer |
|  | PNT-2258  ProNAi Therapeutics | Phase II | Bcl-2 Inhibitors  Signal Transduction Modulators  Apoptosis Inducers | Non-Hodgkin's Lymphoma  Solid Tumors |
|  | Navitoclax  National Cancer Institute  AbbVie | Phase II | Bcl-xl Inhibitors  Bcl-2 Inhibitors  Signal Transduction Modulators  Bcl-2- Related Protein A1 (BFL-1; BCL2A1) Inhibitors  Bcl-w Inhibitors  Apoptosis Inducers | Lung Cancer  Lymphocytic Leukemia  Multiple Myeloma  Prostate Cancer Lymphoma  Solid Tumors  Liver Cancer  Antineoplastic Enhancing Agents |
| BIRC5 | Gataparsen  Isis Pharmaceuticals  Lilly | Phase II | Apoptosis Inducers  BIRC5 (Survivin) Expression  Inhibitors | Prostate Cancer  Non-Small Cell Lung Cancer  Oncolytic Drugs  Myeloid Leukemia |
|  | SVN53-67/M57-KLH  Roswell Park Cancer Institute | Phase II | Cancer Immuno |  |
| CCND1 | Curcumin  Tel Aviv Sourasky Medical Center  Plantacor Central Drug Research  Institute Mahidol University  M.D. Anderson Cancer Center  Johns Hopkins University  Hadassah Medical Organization  Seer Pharmaceuticals  Chinese University of Hong Kong  University of Pennsylvania  University of California, Los Angeles | Phase II | Prostaglandin G/H  Synthase 2 (PTGS2; COX-2)  Inhibitors  CCND1 Expression Inhibitors  NF-kappaB (NFKB) Activation Inhibitors  HIV Integrase Inhibitors  Signal Transduction  Modulators P-Glycoprotein  (MDR-1; ABCB1) Inhibitors  Anti-inflammatory Drugs  AP-1 Inhibitors  Histone N-Acetyltransferase  (HAT) Inhibitors  Glucose-6- phosphatase Inhibitors  Apoptosis Inducers  Antioxidants  Prostaglandin G/H Synthase 1 (PTGS1; COX-1) Inhibitors  DNA Methyltransferase 1 (DNMT1) Inhibitors  Tau Aggregation Inhibitors  EGFR Expression Inhibitors  Angiogenesis Inhibitors  Free Radical Scavengers  Lipoxygenase Inhibitors  FtsZ Inhibitors  Wnt Signaling Inhibitors | Multiple Myeloma  Myelodysplastic Syndrome  Antimalarials  Cystic Fibrosis  Premalignant Conditions  Chemopreventive Agents Treatment of Mucositis  Alzheimer's Dementia,  Pancreatic Cancer  Antiarthritic Drugs  Antipsoriatics  Colorectal Cancer  Antibacterial Drugs  Ocular Genetic  Disorders |
| CDK1 | Palbociclib (Prop INN; USAN), IBRANCE | Launched-2015 | CDK6/Cyclin D3 Inhibitors  CDK4/Cyclin D3 Inhibitors  CDK4 Inhibitors  CDK6 Inhibitors | Lymphocytic Leukemia  Multiple Myeloma  Non-Small Cell  Lung Cancer  Melanoma  Breast Cancer  Myeloid Leukemia  Non-Hodgkin's Lymphoma |
|  | Prazosin Hydrochloride  Pfizer  Centre for Addiction and  Mental Health  Sanofi  Yale University National Institute on Aging | Launched-1974 | CDK1 Inhibitors Signal  Transduction Modulators  alpha1- Adrenoceptor  Antagonists  Apoptosis Inducers | Treatment of Alcohol  Dependency  Mood Disorders,  Benign Prostatic Hyperplasia  Posttraumatic Stress Disorder  (PTSD)  Raynaud's Phenomenon,  Heart Failure  Smoking Cessation Aid |
|  | Rigosertib sodium  Baxter  Nat Heart, Lung, and Blood Institute  TempleUniversity  Onconova  SymBio | Phase III | Phosphatidylinositol  3-Kinase (PI3K) Inhibitors  CDK1 Inhibitors  Signal Transduction Modulators  Apoptosis Inducers  Angiogenesis Inhibitors  Polo-like Kinase-1 (Plk-1) Inhibitors  Antimitotic Drugs | Lymphocytic Leukemia  Myelodysplastic Syndrome  Lymphoma  Ovarian Cancer  Pancreatic Cancer  Myeloid Leukemia  Head and Neck Cancer  Solid Tumors |
|  | Dinaciclib  National Cancer Institute  Merck & Co.  Ligand | Phase III | Transduction Modulators  CDK1/Cyclin B Inhibitors  CDK5/p25 Inhibitors  Breast Cancer- Resistant  Protein (BCRP; ABCG2) Inhibitors  CDK9/Cyclin T1 Inhibitors  Apoptosis Inducers  CDK2/Cyclin A Inhibitors | Lymphocytic Leukemia  Multiple Myeloma  Non-Small Cell  Lung Cancer  Melanoma  Breast Cancer  Myeloid Leukemia  Non-Hodgkin's Lymphoma |
|  | P-276-00  Piramal Life  Sciences | Phase II/III | TNF-alpha Modulators  CDK4/Cyclin D1 Inhibitors  Signal Transduction Modulators  CDK1/Cyclin B Inhibitors  CDK9/Cyclin T1 Inhibitors  Apoptosis Inducers | Multiple Myeloma  Cervical Cancer  Melanoma  Breast Cancer  Mucositis  Pancreatic Cancer  Non-Hodgkin's Lymphoma  Head and Neck Cancer |
|  | 7-Hydroxystaurosporine  National Cancer Institute  Kyowa Hakko Kirin  Keryx | Phase II | Checkpoint Kinase 1 (Chk1) Inhibitors  Checkpoint Kinase 2 (Chk2) Inhibitors  CDK1 Inhibitors Signal Transduction Modulators  Phosphatidylinositol 3-Kinase (PI3K) Inhibitors  Phosphoinositide Dependent Kinase (PDK) 1 Inhibitors  Na+/H+ Exchanger (NHE) Inhibitors  CDK4 Inhibitors  CDK2 Inhibitors  Apoptosis Inducers CDK6 Inhibitors  Protein Kinase C (PKC) Inhibitors | Lymphocytic Leukemia  Small Cell Lung Cancer  Lymphoma  Leukemia  Melanoma  Oncolytic Drugs  Ovarian Cancer  Myeloid Leukemia  Non- Hodgkin's Lymphoma |
|  | Alvocidib  Hydrochloride  National Cancer Institute  Sanofi  Memorial Sloan-Kettering Cancer Center  Tolero  Pharmaceuticals  Mayo Clinic | Phase II | Mcl-1 Inhibitors  Bcl-2 Inhibitors  CDK1 Inhibitors  Signal Transduction Modulators  CDK4 Inhibitors  CDK9/Cyclin T1 Inhibitors  CDK2 Inhibitors  CDK7 Inhibitors  Apoptosis Inducers  CDK6 Inhibitors  Survivin Inhibitors  X-Chromosome- Linked Inhibitor of Apoptosis Protein (XIAP)  Inhibitors | Prostate Cancer  Lymphocytic Leukemia  Multiple Myeloma  Sarcoma  Lung Cancer  Leukemia  Gastric Cancer  Melanoma  Oncolytic Drugs  Breast Cancer  Ovarian Cancer  Cancer of Unspecified Body Location/System  Pancreatic Cancer  Colorectal Cancer  Renal Cancer  Myeloid Leukemia  Hematological Cancer  Liver Cancer  Non-Hodgkin's Lymphoma  Solid Tumors  Head and Neck Cancer |
|  | Roscovitine  Cyclacel  Institute of Cancer Research  (ICR) CNRS | Phase II | CDK1 Inhibitors Signal Transduction Modulators  CDK5 Inhibitors  CDK2 Inhibitors | Oncolytic Drugs |
|  | Seliciclib  Cyclacel  CNRS Institute of Cancer Research  (ICR) | Phase II | CDK9 Inhibitors  Signal Transduction Modulators  CDK1 Inhibitors  CDK5 Inhibitors  CDK2 Inhibitors  CDK7 Inhibitors  Apoptosis Inducers | Lymphocytic Leukemia  MultiplE Myeloma  Cushing's Syndrome,  Non-Small Cell Lung Cancer  Lymphoma  Cystic Fibrosis  Oncolytic Drugs  Breast Cancer  Ovarian Cancer  Rheumatoid Arthritis  Nephritis  Agents for Solid Tumors  Head and Neck Cancer |
|  | AT-7519  Astex Pharmaceuticals  Multiple Myeloma  Research Consortium Novartis  Canadian Cancer Society Research Inst | Phase II | CDK9 Inhibitors  Signal Transduction  Modulators  CDK1/Cyclin B Inhibitors  CDK2/Cyclin A Inhibitors  Apoptosis Inducers | Multiple Myeloma  Leukemia  Non-Hodgkin's Lymphoma  Solid Tumors |
|  | Milciclib  Nerviano Medical Sciences  TGen Research InstitutePfizer  Tiziana Life Sciences  Johns Hopkins University | Phase II | CDK1 Inhibitors  Signal Transduction Modulators  CDK4 Inhibitors  CDK5 Inhibitors  CDK2 Inhibitors  High Affinity Nerve Growth  Factor Receptor (TrKA)  Inhibitors  CDK2/Cyclin A Inhibitors | Respiratory/Thoracic Cancer  Oncolytic Drugs  Breast Cancer  Liver Cancer  Solid Tumors |
|  | BAY-1000394  Bayer |  | Aurora-A (ARK1) Kinase  Inhibitors  VEGFR-3 (FLT4) Inhibitors  MAP3K9 (MLK1) Inhibitors  CDK4/Cyclin D1 Inhibitors  Signal Transduction Modulators  CDK1/Cyclin B Inhibitors  Jak3 Inhibitors  CDK9/Cyclin T1 Inhibitors  Jak2 Inhibitors  AngiogenesisMInhibitors  CDK2/Cyclin E Inhibitors  CDK3/Cyclin E Inhibitors  Antimitotic Drugs | Small Cell Lung Cancer  Ovarian Cancer  Solid Tumors |
| DTL | Datelliptium chloride  BPI-Groupe  Sanofi  CNRS | Phase III | Alkaloids |  |
| EGFR | Quercetin    Molsoft  Cincinnati  Children's Hospital Med Cent  Limerick BioPharma  Guizhou University | Launched | alpha- Glucosidase Inhibitors  MAO-A Inhibitors  Nav1.5 (Cardiac/SkMII)  Sodium Channel Blockers  NADDependent Protein Deacetylase Sirtuin-(SIRT1) Activators  Signal Transduction Modulators  Xanthine Oxidase Inhibitors  Cytokine Production Inhibitors  EGFR (HER1; erbB1) Inhibitors  Drugs Acting on Quorum Sensing Signaling Antioxidants  Aldose Reductase Inhibitors  Protein Tyrosine Phosphatase PTP-1B Inhibitors  Wnt Signaling Inhibitors  Free Radical Scavengers | Hemostatics  Antianemics  Non-Opioid Analgesics  Cardioprotectants  Oncolytic Drugs  Angina pectoris,  Antidiabetic Drugs  Antibacterial Drugs  Symptomatic Antidiabetic Agents |
|  | Cetuximab  National Cancer Institute  Merck  Serono Universitaet zu Koeln  Vanderbilt University  Merck  KGaA  Lilly  Bristol- Myers Squibb  University College London  National Taiwan University  Universityof Michigan | Launched-2003 | Signal Transduction Modulators  P-Glycoprotein (MDR-1;  ABCB1) Inhibitors  Angiogenesis Inhibitors  Anti-EGFR | Respiratory/Thoracic Cancer  Prostate Cancer  Multiple Myeloma  Non-Small Cell Lung Cancer  Cervical Cancer  Neuropathic Pain,  Gastric Cancer  Bladder Cancer  Breast Cancer  Ovarian Cancer  Digestive/Gastrointestinal Cancer  Pancreatic Cancer  Colorectal Cancer  Renal Cancer  Head and Neck Cancer  Liver Cancer |
|  | Gefitinib  National Cancer Institute  Stanford University  EORTC  AstraZeneca  M.D. Anderson Cancer Center  Dana-Farber Cancer Institute  Canadian Cancer Society Research Inst  University of Nebraska  St Jude Children's Research Hospital | Launched-2002 | Signal Transduction Modulators  EGFR (HER1; erbB1) Inhibitors | Small Cell Lung Cancer  Prostate Cancer  Sarcoma  Non-Small Cell Lung Cancer  Endocrine Cancer  Astrocytoma  Neurologic Cancer  Gastric Cancer  Bladder Cancer  Breast Cancer  Ovarian Cancer  Cancer of Unspecified Body Location/System  Pancreatic Cancer  Colorectal Cancer  Glioblastoma Multiforme  Myeloid Leukemia  Renal Cancer  Squamous Cell Carcinoma  Head and Neck Cancer  Solid Tumors  Liver Cancer |
|  | Erlotinib Hydrochloride  National Cancer Institute  Genentech  EORTC  Hopitaux Universitaires de Strasbourg  Roche  Pfizer  Chugai Pharmaceutical  M.D. Anderson Cancer Center  University of California, San Francisco  Mayo Clinic  Astellas Pharma  National Cancer Research Institute  University of California, Davis  Sanofi  Dana-Farber Cancer Institute  Schwarz Pharma  Canadian Cancer Society Research Inst | Launched-2004 | Signal Transduction Modulators EGFR (HER1; erbB1) Inhibitors | Prostate Cancer  Myelodysplastic Syndrome  Sarcoma  Non-Small Cell Lung Cancer  Premalignant Conditions  Gastrointestinal  Astrocytoma  Cervical Cancer  Neurologic Cancer  Gastric Cancer  Melanoma  Agents for Viral Hepatitis  Bladder Cancer  Brain Cancer  Breast Cancer  Ovarian Cancer  Digestive/Gastrointestinal Cancer  Pancreatic Cancer  Colorectal Cancer  Renal Cancer  Glioblastoma Multiforme  Myeloid Leukemia  Hematological Cancer  Head and Neck Cancer  Solid Tumors  Liver Cancer |
|  | Panitumumab  Takeda National Cance rInstitute  Amgen | Launched-2006 | Signal Transduction Modulators Anti-EGFR Human Monoclonal  Antibodies | Prostate Cancer  Non-Small Cell Lung Cancer  Breast Cancer  Ovarian Cancer  Digestive/Gastrointestinal  Cancer  Pancreatic Cancer  Colorectal Cancer  Renal Cancer  Head and Neck Cancer |
|  | Nimotuzumab  BioTech Pharmaceutical  Kuhnil Pharmaceutical  CIMAB  InnoMab Te Arai  BioFarma  Oncoscience  Daiichi Sankyo  Gilead  Eurofarma Laboratorios  Innogene  Biocon | Launched-2006 | Signal Transduction Modulators  Anti-EGFR | Prostate Cancer  Non-Small Cell Lung Cancer  Astrocytoma  Cervical Cancer  Neurologic Cancer  Gastric Cancer  Brain Cancer  Breast Cancer  Digestive/Gastrointestinal Cancer  Pancreatic Cancer  Colorectal Cancer  Glioblastoma Multiforme  Head and Neck Cancer  Solid Tumors  Liver Cancer |
|  | Lapatinib ditosylate  National Cancer Institute  EORTC  Novartis  GlaxoSmithKline  M.D. Anderson Cancer Center  Concert Pharmaceuticals  Brown University  Cedars-Sinai Medical Center  Mayo Clinic | Launched-2007 | Signal Transduction Modulators  EGFR (HER1; erbB1) Inhibitors  HER2 (erbB2) Inhibitors | Prostate Cancer  Endocrine Cancer  Neurological Genetic  Disorders  Neurologic Cancer  Cervical Cancer  Lung Cancer  Gastric Cancer  Bladder Cancer  Breast Cancer  Ovarian Cancer  Digestive/Gastrointestinal  Cancer  Cancer of Unspecified Body Location/System  Pancreatic Cancer  Colorectal Cancer  Renal Cancer  Glioblastoma Multiforme  Liver Cancer  Non-Hodgkin's Lymphoma  Head and Neck Cancer |
|  | Bosutinib  Pfizer | Launched-2012 | Bcr-Abl (Bcr- Abl1) Kinase  Inhibitors  Signal Transduction Modulators  Src Kinase Inhibitors  Signal Transducer and Activator of Transcription  5 (STAT5) Inhibitors  Apoptosis Inducers  Abl1 Kinase Inhibitors | Treatment of  Renal  Diseases  Non-Small Cell Lung Cancer  Leukemia  Ischemic Stroke  Breast Cancer  Pancreatic Cancer  Colorectal Cancer  Glioblastoma Multiforme  Myeloid Leukemia |
|  | Vandetanib  National Cancer Institute  Genzyme  AstraZeneca  M.D. Anderson Cancer Center  Dana- Farber Cancer Institute  Cardiff University | Launched-2011 | VEGFR-2 (FLK-1/KDR) Inhibitors  VEGFR-3 (FLT4) Inhibitors  Signal Transduction Modulators  KIT (C-KIT) Inhibitors  RET Inhibitors  EGFR (HER1; erbB1) Inhibitors  Flt3 (FLK2/STK1) Inhibitors  Angiogenesis Inhibitors  VEGFR-1 (Flt-1) Inhibitors  Abl Kinase Inhibitors | Respiratory/Thoracic Cancer  Prostate Cancer  Non-Small Cell Lung Cancer  Endocrine Cancer  Neurological Genetic Disorders Neurologic Cancer  Breast Cancer  Bladder Cancer  Digestive/Gastrointestinal Cancer  Pancreatic Cancer  Female Reproductive  System Cancer  Colorectal Cancer  Renal Cancer  Glioblastoma Multiforme  Genitourinary Cancer  Cancer Associated  Disorders, Treatment of  Head and Neck Cancer  Liver Cancer |
|  | Afatinib  National Cancer Institute  Johannes Gutenberg- Universitaet  Mainz  Boehringer Ingelheim  Nippon  Boehringer Ingelheim | Launched-2013 | Signal Transduction Modulators  EGFR (HER1; erbB1) Inhibitors  HER4 (erbB4) Inhibitors  HER2 (erbB2)Inhibitors | Prostate Cancer  Non-Small Cell Lung Cancer  Neurologic Cancer  Gastric Cancer  Bladder Cancer  Breast Cancer  Digestive/Gastrointestinal  Cancer  Pancreatic Cancer  Female Reproductive  System Cancer  Colorectal Cancer  Glioblastoma Multiforme  Head and Neck Cancer |
|  | Tivozanib  Kyowa Hakko  Kirin  AVEO Pharma  Astellas Pharma  Emory University  Pharmstandard  General  Hospital Corp.  Northwest University | Phase III | VEGFR-2 (FLK-1/KDR) Inhibitors  VEGFR-3 (FLT4) Inhibitors  Signal Transduction Modulators  VEGFR-1 (Flt-1) Inhibitors  Angiogenesis Inhibitors  Tyrosine Kinase Inhibitors | Sarcoma  Age-Related Macular  Degeneration  Non-Small Cell Lung Cancer  Astrocytoma  Oncolytic Drugs  Breast Cancer  Ovarian Cancer  Female Reproductive  System Cancer  Colorectal Cancer  Renal Cancer  Solid Tumors  Liver Cancer |
|  | Neratinib  Pfizer  Dana-Farber Cancer  Institute  Puma Biotechnology | Phase III | Signal Transduction Modulators  EGFR (HER1; erbB1) Inhibitors  HER4 (erbB4) Inhibitors  HER2 (erbB2) Inhibitors | Non-Small Cell  Lung Cancer  Breast Cancer  Solid Tumors |
|  | Dovitinib lactate  Novartis  Samsung Medical Center | Phase III | VEGFR-2 (FLK-1/KDR) Inhibitors  PDGFRbeta Inhibitors  FGFR3 Inhibitors  Signal Transduction Modulators  EGFR (HER1; erbB1) Inhibitors  VEGFR-1 (Flt-1) Inhibitors  Angiogenesis Inhibitors  FGFR1 Inhibitors | Respiratory/Thoracic Cancer  Multiple Myeloma  Prostate Cancer  Non-Small Cell Lung Cancer  Endocrine Cancer  Neurological Genetic  Disorders  Gastric Cancer  Melanoma  Breast Cancer  Bladder Cancer  Female Reproductive  System Cancer  Pancreatic Cancer  Digestive/Gastrointestinal  Cancer  Colorectal Cancer  Renal Cancer  Glioblastoma Multiforme  Myeloid Leukemia  Solid Tumors  Liver Cancer  Head and Neck Cancer |
|  | Tesevatinib  Symphony Evolution  Kadmon  Exelixis | Phase III | VEGFR-2 (FLK-1/KDR) Inhibitors  VEGFR-3 (FLT4) Inhibitors  Signal Transduction Modulators  EGFR (HER1; erbB1) Inhibitors  Src Kinase Inhibitors  Angiogenesis Inhibitors  HER2 (erbB2) Inhibitors  EphB4Inhibitors | Renal  Diseases  Non-Small Cell  Lung Cancer  Breast Cancer |
|  | Zalutumumab | Phase III | Transduction Modulators  Anti-EGFR | Non-Small Cell Lung Cancer  Colorectal Cancer  Head and Neck Cancer |
|  | Necitumumab  MedImmune  Dyax  Merck KGaA  Lilly | Pre-Registered | Signal Transduction Modulators  Anti-EGFR | Non-Small Cell Lung Cancer  Colorectal Cancer  Solid Tumors |
|  | Dacomitinib  SFJ Pharmaceuticals  Pfizer | Phase III | Signal Transduction Modulators  EGFR (HER1; erbB1) Inhibitors  HER4 (erbB4) Inhibitors  HER2 (erbB2) Inhibitors | Non-Small Cell Lung Cancer  Brain Cancer  Glioblastoma Multiforme  Head and Neck Cancer  Solid Tumors  Squamous Cell Carcinoma |
|  | Tivantinib  National Cancer Institute  ArQule  Kyowa Hakko Kirin  Dana-Farber Cancer Institute  Daiichi Sankyo | Phase III | Signal Transduction Modulators  Apoptosis Inducers  HGFR (MET; c-Met) Inhibitors | Prostate Cancer  Non-Small Cell Lung Cancer  Gastric Cancer  Breast Cancer  Cancer of Unspecified Body  Location/System  Pancreatic Cancer  Colorectal Cancer  Renal Cancer  Solid Tumors  Liver Cancer |
|  | Icotinib Hydrochloride  Guangdong General Hospital  Beta Pharma (US) | Launched-2011 | Signal Transduction Modulators  EGFR (HER1; erbB1) Inhibitors | Non-Small Cell Lung Cancer  Brain Cancer  Pancreatic Cancer  Digestive/Gastrointestinal  Cancer  Antipsoriatics  Head and Neck Cancer |
|  | Cetuximab  Shanghai National Eng Res Cent AntibMed  Shanghai Biomabs Pharmaceuticals | Phase II/III | Signal Transduction Modulators  Anti-EGFR | Colorectal Cancer |
|  | Osimertinib mesylate  AstraZeneca | Pre-Registered | Signal Transduction Modulators  EGFR (Thr790Met Mutant)  Inhibitors | Non-Small Cell Lung Cancer  Solid Tumors |
|  | Rociletinib Hydrobromide  Celgene  Clovis Oncology | Pre-Registered | Signal Transduction Modulators  EGFR (Thr790Met Mutant)  Inhibitors  Apoptosis Inducers | Non-Small Cell Lung Cancer |
|  | ASP-8273  Astellas Pharma | Phase III | Signal Transduction Modulators  EGFR (HER1; erbB1) Inhibitors  EGFR (Thr790Met Mutant) Inhibitors | Non-Small Cell Lung Cancer |
| ERBB3 | Elisidepsin  PharmaMar | Phase II | Signal Transduction Modulators  HER3 (erbB3) Inhibitors | Non-Small Cell Lung Cancer  Digestive/Gastrointestinal Cancer |
|  | Sapitinib  AstraZeneca | Phase II | Signal Transduction Modulators  EGFR (HER1; erbB1) Inhibitors  HER3 (erbB3) Inhibitors  HER2 (erbB2) Inhibitors | Non-Small Cell Lung Cancer  Gastric Cancer  Breast Cancer  Colorectal Cancer  Solid Tumors |
|  | A5-linker-ML3.9 bispecific scFv  Merrimack  Fox Chase Cancer Center | Phase II | Signal Transduction Modulators  Anti- HER2/neu/ErbB2  Anti-Receptor Tyrosine-Protein  Kinase ErbB-3 (HER3) | Gastric Cancer  Breast Cancer  Digestive/Gastrointestinal  Cancer  Solid Tumors |
|  | Seribantumab  Merrimack | Phase II | Signal Transduction Modulators  Anti-Receptor Tyrosine-  Protein Kinase ErbB-3 (HER3) | Non-Small Cell Lung Cancer  Breast Cancer  Oncolytic Drugs  Ovarian Cancer  Female Reproductive  System Cancer  Solid Tumors |
|  | Patritumab  U3 Pharma  Amgen  Daiichi Sankyo | Phase III | Signal Transduction Modulators  Anti-Receptor Tyrosine-  Protein Kinase ErbB-3 (HER3) | Non-Small Cell Lung Cancer  Oncolytic Drugs  Breast Cancer  Head and Neck Cancer |
|  | MM-141  Merrimack | Phase II | Signal Transduction Modulators  Anti-CD221(IGF-1R)  Anti-Receptor Tyrosine-  Protein Kinase ErbB-3 (HER3) | Pancreatic Cancer  Solid Tumors |
|  | Duligotuzumab  Genentech | Phase II | Signal Transduction Modulators  Anti-EGFR Anti-Receptor  Tyrosine- Protein Kinase  ErbB-3 (HER3) | Colorectal Cancer  Solid Tumors  Head and Neck Cancer |
| ERBB4 | Canertinib  Dihydrochloride  Pfizer | Phase II | Transduction Modulators  EGFR (HER1; erbB1) Inhibitors  HER4 (erbB4) Inhibitors  HER2 (erbB2) Inhibitors | Non-Small Cell  Lung Cancer  Breast Cancer |
|  | Afatinib  National Cancer Institute  Johannes Gutenberg- Universitaet  Mainz  Boehringer Ingelheim  Nippon  Boehringer Ingelheim | Launched-2013 | Signal Transduction Modulators  EGFR (HER1; erbB1) Inhibitors  HER4 (erbB4) Inhibitors  HER2 (erbB2) Inhibitors | Prostate Cancer  Non-Small Cell Lung Cancer  Neurologic Cancer  Gastric Cancer  Bladder Cancer  Breast Cancer  Digestive/Gastrointestinal  Cancer  Pancreatic Cancer  Female Reproductive System Cancer  Colorectal Cancer  Glioblastoma Multiforme  Head and Neck Cancer |
|  | Neratinib  Pfizer  Dana-Farber Cancer Institute  Puma Biotechnology | Phase III | Signal Transduction Modulators  EGFR (HER1; erbB1) Inhibitors  HER4 (erbB4) Inhibitors  HER2 (erbB2) Inhibitors | Non-Small Cell Lung Cancer  Breast Cancer  Solid Tumors |
|  | Dacomitinib  SFJ Pharmaceuticals  Pfizer | Phase III | Transduction Modulators  EGFR (HER1; erbB1) Inhibitors  HER4 (erbB4) Inhibitors  HER2 (erbB2) Inhibitors | Non-Small Cell Lung Cancer  Brain Cancer  Glioblastoma Multiforme  Head and Neck Cancer  Solid Tumors  Squamous Cell Carcinoma |
|  | BMS-690514  Bristol-Myers Squibb | Phase II | VEGFR-3 (FLT4)  Inhibitors  VEGFR-2 (FLK-1/KDR)  Inhibitors  Signal Transduction Modulators  HER4 (erbB4) Inhibitors  EGFR (HER1; erbB1)  Inhibitors  VEGFR-1 (Flt-1) Inhibitors  Angiogenesis Inhibitors  HER2 (erbB2) Inhibitors | Non-Small Cell Lung Cancer  Breast Cancer  Solid Tumors |
|  | Poziotinib  Spectrum Pharmaceuticals  Luye Pharma  Hanmi | Phase II | Signal Transduction  Modulators  EGFR (HER1; erbB1) Inhibitors  HER4 (erbB4) Inhibitors  HER2 (erbB2) Inhibitors | Non-Small Cell Lung Cancer  Gastric Cancer  Breast Cancer  Head and Neck Cancer  Solid Tumors |
|  | Tarloxotinib bromide  Proacta  University of Auckland  Yakult Honsha  Threshold Pharmaceuticals | Phase II | Signal Transduction  Modulators  EGFR (HER1; erbB1)  Inhibitors  HER4 (erbB4) Inhibitors  HER2 (erbB2) Inhibitors | Non-Small Cell Lung Cancer  Skin Cancer  Head and Neck Cancer |
| FGF18 | Sprifermin | Phase II |  | Cartilage Disorders,  Treatment of Osteoarthritis  Treatment of Antiarthritic |
| GSK3B | Cycloheximide  University of Hawaii  University of Minnesota  Pfizer  Universiti Putra  Malaysia | Launched | Signal Transduction  Modulators  Glycogen Synthase  Kinase 3 beta (GSK-3beta;  tau Protein Kinase I) Inhibitors | Oncolytic Drugs  Antifungal Agents |
|  | LY-2090314  H. Lee Moffitt Center  Lilly | Phase I/II | Signal Transduction Modulators  Glycogen Synthase Kinase 3 beta(GSK-3beta; tau Protein  Kinase I) Inhibitors | Leukemia  Oncolytic Drugs  Pancreatic Cancer  Type 2 Diabetes |
| MDM2 | AMG-232  Amgen | Phase I/II | MDM2 (hdm2) Inhibitors | Melanoma  Myeloid Leukemia  Solid Tumors |
|  | ALRN-6924  Roche  Aileron Therapeutics | Phase I/II | MDM4 (MDMX) Inhibitors  MDM2 (hdm2) Inhibitors | Oncolytic Drugs  Hematological Cancer  Solid Tumors |
|  | HDM-201  Novartis | Phase I/II | MDM2 (hdm2) Inhibitors | Sarcoma  Hematological Cancer  Solid Tumors |
| MMP9 | Zoledronic acid Monohydrate  Novartis  Merrion  University of Alabama at Birmingham  University of California, San Francisco  Axsome Therapeutics  Universiteit Leiden  Thar Pharmaceuticals  Asahi Kasei | Launched-2000 | Drugs Targeting Tumor- Associated Macrophages  Farnesyl Pyrophosphate Synthase Inhibitors  MMP9 Expression Inhibitors  Angiogenesis Inhibitors | Bone Cancer  Prostate Cancer  Treatment of Paget's Disease  Neurologic Cancer  Bone Resorption Inhibitors  Premalignant Conditions  Neuropathic Pain,  Oncolytic Drugs  Breast Cancer  Rheumatoid Arthritis  Osteoporosis  Sickle Cell Anemia  Hypercalcemia  Solid Tumors  Bone Diseases |
|  | Teriflunomide  Sanofi  Sugen  Genzyme | Launched-2012 | MMP9 Expression Inhibitors  MMP-9 (Gelatinase B) Inhibitors  Dihydroorotate Dehydrogenase (DHODH) Inhibitors  MMP-2 (Gelatinase A) Inhibitors | Disease-Modifying Anti-Rheumatic Drugs Immunosuppressants  Multiple Sclerosis |
| NAT1 | Mesalazine  Aptalis Shire Mochida  Giuliani  Warner  Chilcott  Abbott  Astellas Pharma  Sanofi  Gentium  Falk  Pharma  Tillotts  SOFAR  Merckle  Recordati  Kyorin  Kyowa  Hakko Kirin  Cosmo  Salix  Zeria  Ajinomoto  Meda  Karolinska Institutet  Ferring | Launched-1984 | Protein Phosphatase 2A (PP-2A) Inhibitors  Arylamine N-acetyltransferase 1 (NAT1) Inhibitors  Signal Transduction Modulators beta-Catenin Inhibitors | Inflammatory Bowel Disease,  Gastrointestinal Disorders (Not  Specified)  Irritable Bowel Syndrome, |
| PLK1 | Rigosertib sodium  Baxter  Nat Heart, Lung, and Blood Institute  Temple University  Onconova  SymBio | Phase III | Phosphatidylinositol 3-Kinase (PI3K) Inhibitors  CDK1 Inhibitors  Signal Transduction Modulators  Apoptosis Inducers  Angiogenesis Inhibitors  Polo-like Kinase-1 (Plk-1) Inhibitors  Antimitotic Drugs | Lymphocytic Leukemia  Myelodysplastic Syndrome  Lymphoma  Ovarian Cancer  Pancreatic Cancer  Myeloid Leukemia  Head and Neck Cancer  Solid Tumors |
|  | Volasertib  Boehringer Ingelheim | Phase III | Signal Transduction Modulators  Polo-like Kinase-1 (Plk-1) Inhibitors  Antimitotic Drugs | Non-Small Cell Lung Cancer  Bladder Cancer  Ovarian Cancer  Oncolytic Drugs  Female Reproductive System Cancer  Myeloid Leukemia |
|  | PLK1-SNALP  Arbutus Biopharma  Alnylam Pharmaceuticals | Phase II | PLK1 Expression Inhibitors | Lymphoma  Endocrine Cancer  Solid Tumors  Liver Cancer |
| RRM2 | LOR-2040  National Cancer Institute  Aptose Biosciences | Phase II | RRM2 Expression Inhibitors | Prostate Cancer  Myelodysplastic Syndrome  Non-Small Cell Lung Cancer  Lymphoma  Leukemia  Bladder Cancer  Breast Cancer  Colorectal Cancer  Renal Cancer  Myeloid Leukemia |
| TGFb | Fresolimumab  National Cancer Institute  Icahn School of Medicine at Mount Sinai  Genzyme  MedImmune  Sanford- Burnham Medical  Research Inst  University of Pennsylvania  Boston University | Phase II | Anti- TGFbeta2  Signal Transduction Modulators Anti- TGFbeta  Anti- TGFbeta3 | Interstitial Lung Diseases,  Renal Diseases  Respiratory/Thoracic Cancer  Non-Small Cell Lung Cancer  Scleroderma  Neurologic Cancer  Melanoma  Renal Cancer  Solid Tumors  Hematopoiesis Disorders |

Compound search conducted using Thomson Reuters Integrity℠

***Supplementary Table 7: Coefficients and P-values of mRNA-IHC4 Risk Model***

|  | **exp(coef)** | **exp(-coef)** | **lower .95** | **upper .95** |  |
| --- | --- | --- | --- | --- | --- |
| **ESR1** | 1.03637 | 0.96490 | 0.88913 | 1.20801 |  |
| **HER2** | 1.11903 | 0.89363 | 0.94665 | 1.32279 |  |
| **PGR** | 0.83413 | 1.19885 | 0.74507 | 0.93384 |  |
| **MKI67** | 1.66025 | 0.60232 | 1.26213 | 2.18394 |  |
|  |  |  |  |  |  |
|  |  |  |  |  |  |
|  | **coef** | **exp(coef)** | **se(coef)** | **z** | **Pr(>\|z\|)** |
| **ESR1** | 0.03573 | 1.03637 | 0.07819 | 0.45695 | 0.647705369 |
| **HER2** | 0.11246 | 1.11903 | 0.08535 | 1.31762 | 0.187629665 |
| **PGR** | -0.18136 | 0.83413 | 0.05761 | -3.14801 | 0.001643889 |
| **MKI67** | 0.50697 | 1.66025 | 0.13988 | 3.62424 | 0.000289815 |

**Supplementary References**

1. Schemper,M. & Smith,T.L. A note on quantifying follow-up in studies of failure time. *Control Clin. Trials* 17, 343-346 (1996).

2. Starmans,M.H., Pintilie,M., John,T., Der,S.D., Shepherd,F.A., Jurisica,I. *et al.* Exploiting the noise: improving biomarkers with ensembles of data analysis methodologies. *Genome Med.* 4, 84 (2012).

3. Waggott,D., Chu,K., Yin,S., Wouters,B.G., Liu,F.F., & Boutros,P.C. NanoStringNorm: an extensible R package for the pre-processing of NanoString mRNA and miRNA data. *Bioinformatics.* 28, 1546-1548 (2012).

4. Breitling,R., Armengaud,P., Amtmann,A., & Herzyk,P. Rank products: a simple, yet powerful, new method to detect differentially regulated genes in replicated microarray experiments. *FEBS Lett.* 573, 83-92 (2004).

5. Barton,S., Zabaglo,L., A'Hern,R., Turner,N., Ferguson,T., O'Neill,S. *et al.* Assessment of the contribution of the IHC4+C score to decision making in clinical practice in early breast cancer. *Br. J. Cancer* 106, 1760-1765 (2012).

6. Cuzick,J., Dowsett,M., Pineda,S., Wale,C., Salter,J., Quinn,E. *et al.* Prognostic value of a combined estrogen receptor, progesterone receptor, Ki-67, and human epidermal growth factor receptor 2 immunohistochemical score and comparison with the Genomic Health recurrence score in early breast cancer. *J. Clin. Oncol.* 29, 4273-4278 (2011).

7. Paik,S., Shak,S., Tang,G., Kim,C., Baker,J., Cronin,M. *et al.* A multigene assay to predict recurrence of tamoxifen-treated, node-negative breast cancer. *N. Engl. J. Med.* 351, 2817-2826 (2004).

8. Parker,J.S., Mullins,M., Cheang,M.C., Leung,S., Voduc,D., Vickery,T. *et al.* Supervised risk predictor of breast cancer based on intrinsic subtypes. *J. Clin. Oncol.* 27, 1160-1167 (2009).

9. Chia,S.K., Bramwell,V.H., Tu,D., Shepherd,L.E., Jiang,S., Vickery,T. *et al.* A 50-gene intrinsic subtype classifier for prognosis and prediction of benefit from adjuvant tamoxifen. *Clin. Cancer Res.* 18, 4465-4472 (2012).

10. Nielsen,T.O., Parker,J.S., Leung,S., Voduc,D., Ebbert,M., Vickery,T. *et al.* A comparison of PAM50 intrinsic subtyping with immunohistochemistry and clinical prognostic factors in tamoxifen-treated estrogen receptor-positive breast cancer. *Clin. Cancer Res.* 16, 5222-5232 (2010).

11. Perou,C.M., Sorlie,T., Eisen,M.B., van de Rijn,M., Jeffrey,S.S., Rees,C.A. *et al.* Molecular portraits of human breast tumours. *Nature* 406, 747-752 (2000).

12. van de Vijver,M.J., He,Y.D., van't Veer,L.J., Dai,H., Hart,A.A., Voskuil,D.W. *et al.* A gene-expression signature as a predictor of survival in breast cancer. *N. Engl. J. Med.* 347, 1999-2009 (2002).

13. Toussaint,J., Sieuwerts,A.M., Haibe-Kains,B., Desmedt,C., Rouas,G., Harris,A.L. *et al.* Improvement of the clinical applicability of the Genomic Grade Index through a qRT-PCR test performed on frozen and formalin-fixed paraffin-embedded tissues. *BMC. Genomics* 10, 424 (2009).

14. Sparano,J.A. TAILORx: trial assigning individualized options for treatment (Rx). *Clin. Breast Cancer* 7, 347-350 (2006).

15. Sparano,J.A., Gray,R.J., Makower,D.F., Pritchard,K.I., Albain,K.S., Hayes,D.F. *et al.* Prospective Validation of a 21-Gene Expression Assay in Breast Cancer. *N. Engl. J. Med.*(2015).
