# Supplementary figures and images for: Molecular stratification of early breast cancer identifies drug targets to drive stratified medicine
Source: NPJ Breast Cancer. 2017 Feb 15;3:3. doi: 10.1038/s41523-016-0003-5 (PMC5445616; doi:10.1038/s41523-016-0003-5)

## Slide 1
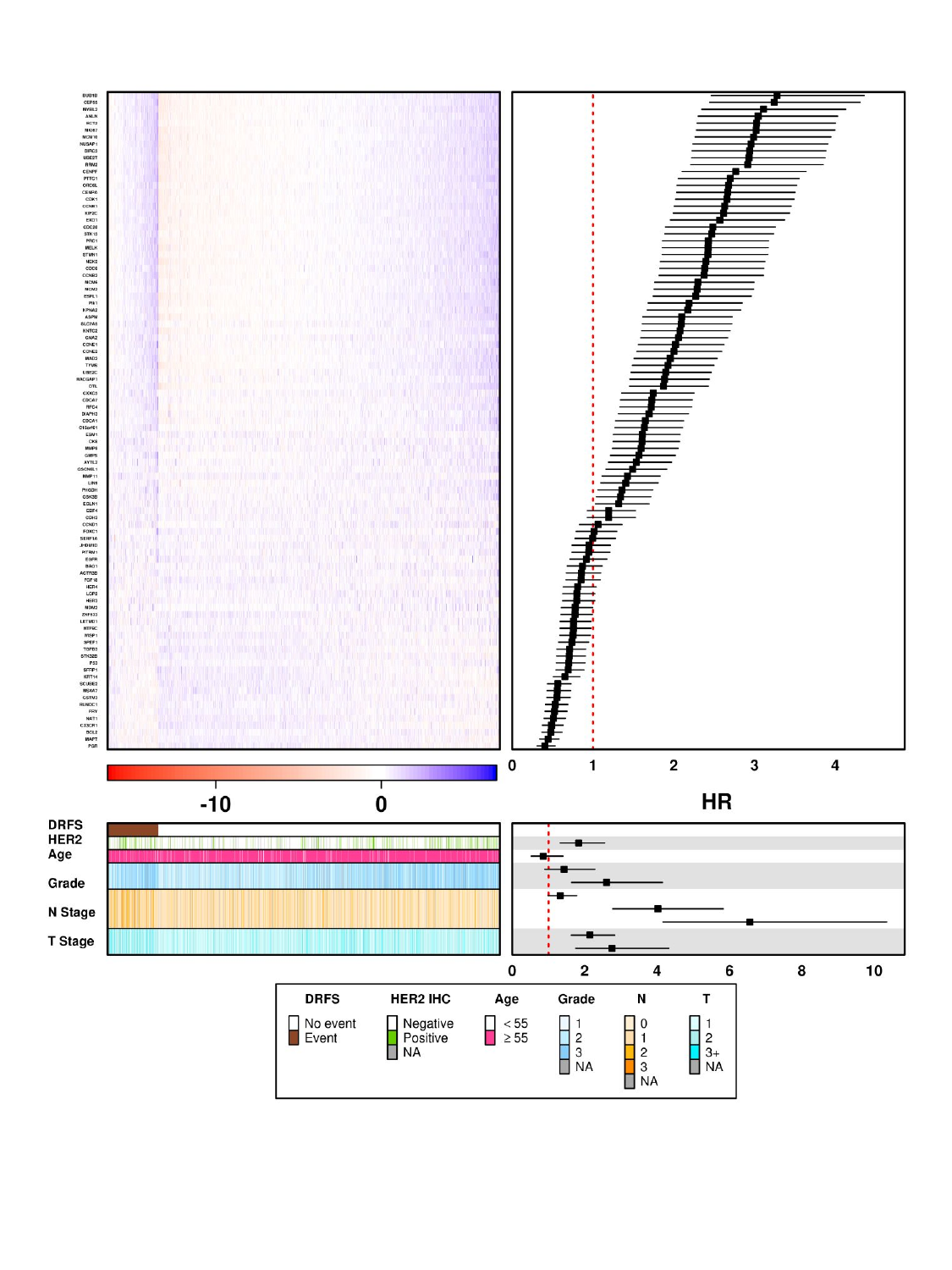

Supplement: Supplementary file 2 — Supplementary Figure 1 [file 41523_2016_3_MOESM2_ESM.pptx]

## Slide 1
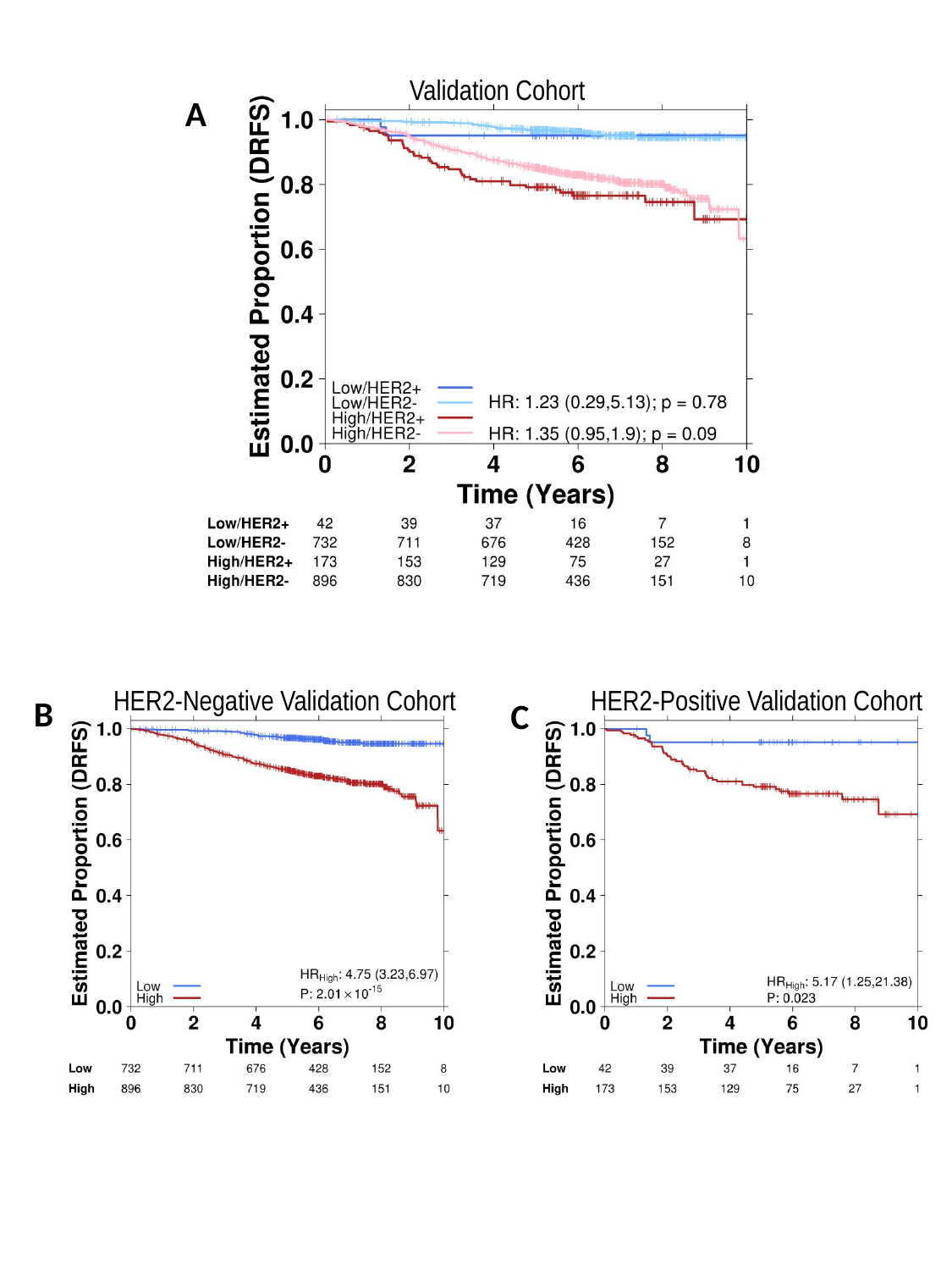

Validation Cohort
A
HER2-Negative Validation Cohort
HER2-Positive Validation Cohort
B
C

Supplement: Supplementary file 6 — Supplementary Figure 5 [file 41523_2016_3_MOESM6_ESM.pptx]

## Slide 1
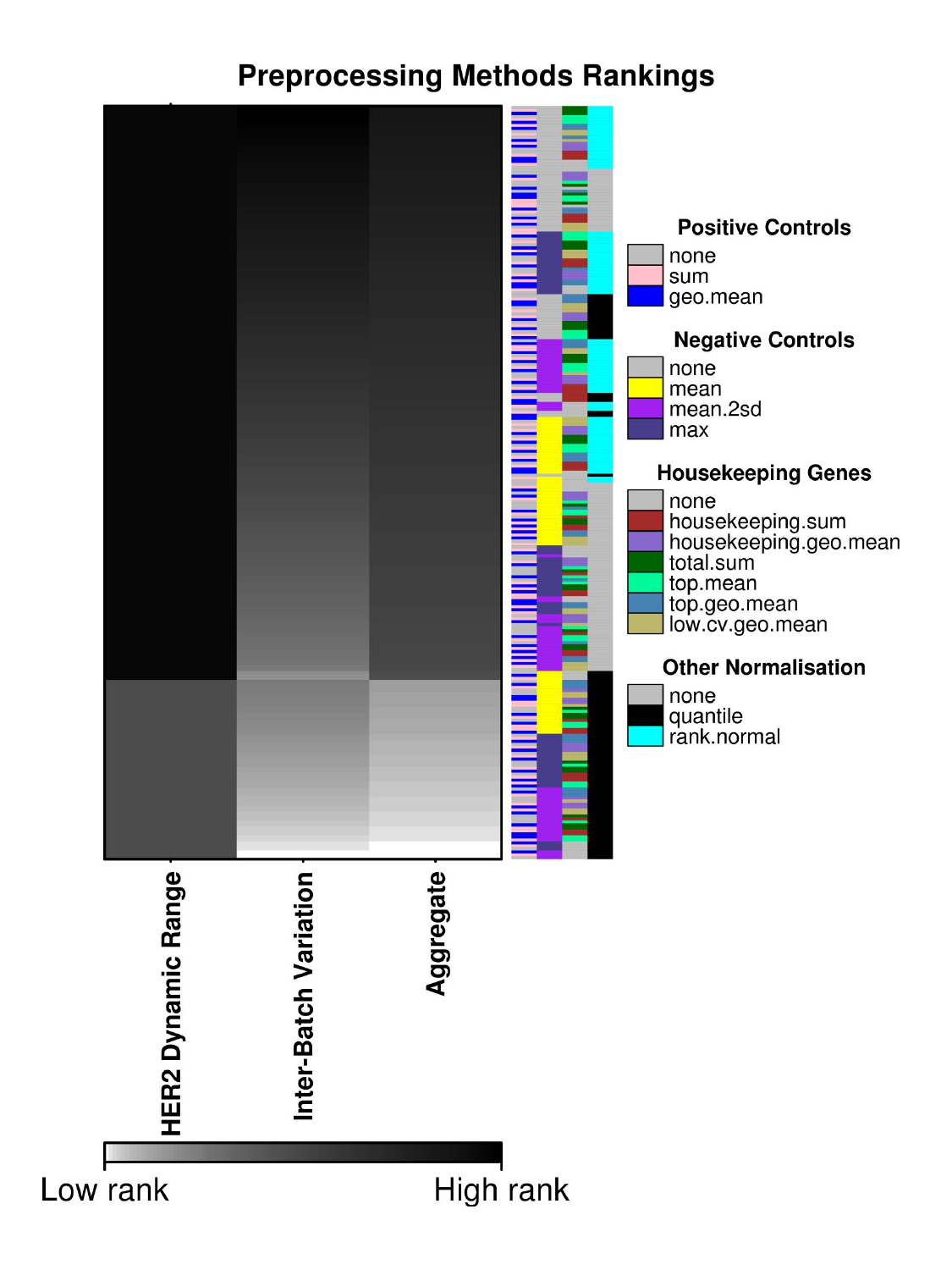

Supplement: Supplementary file 10 — Supplementary Figure 9 [file 41523_2016_3_MOESM10_ESM.pptx]
