# Supplementary material for: Molecular stratification of early breast cancer identifies drug targets to drive stratified medicine
Source: NPJ Breast Cancer. 2017 Feb 15;3:3. doi: 10.1038/s41523-016-0003-5 (PMC5445616; doi:10.1038/s41523-016-0003-5)
Supplement: Supplementary file 4 — Supplementary Figure 3 [file 41523_2016_3_MOESM4_ESM.pptx]

## Slide 1
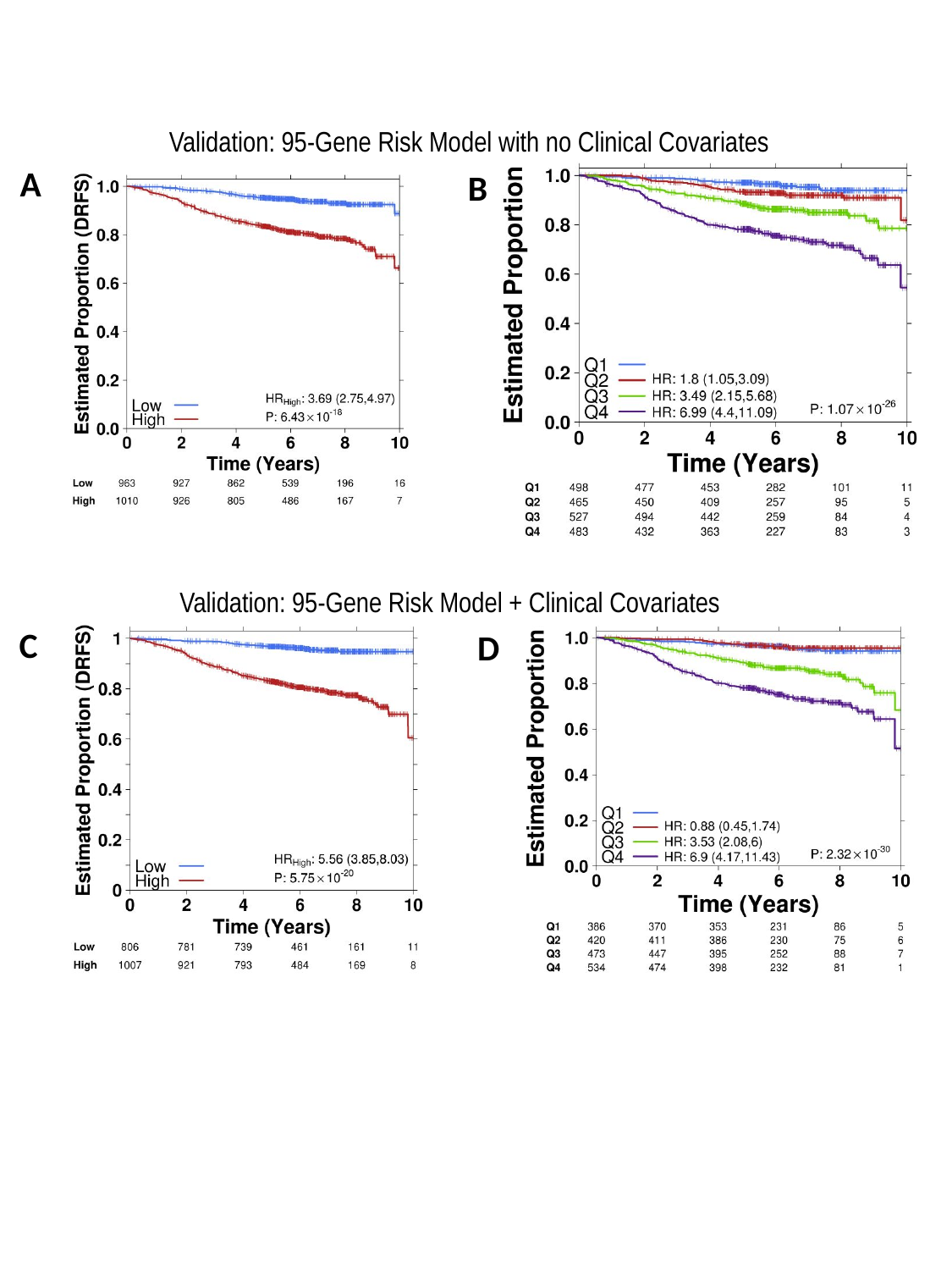

Validation: 95-Gene Risk Model with no Clinical Covariates
A
B
Validation: 95-Gene Risk Model + Clinical Covariates
C
D
