# Supplementary material for: Molecular stratification of early breast cancer identifies drug targets to drive stratified medicine
Source: NPJ Breast Cancer. 2017 Feb 15;3:3. doi: 10.1038/s41523-016-0003-5 (PMC5445616; doi:10.1038/s41523-016-0003-5)
Supplement: Supplementary file 5 — Supplementary Figure 4 [file 41523_2016_3_MOESM5_ESM.pptx]

## Slide 1
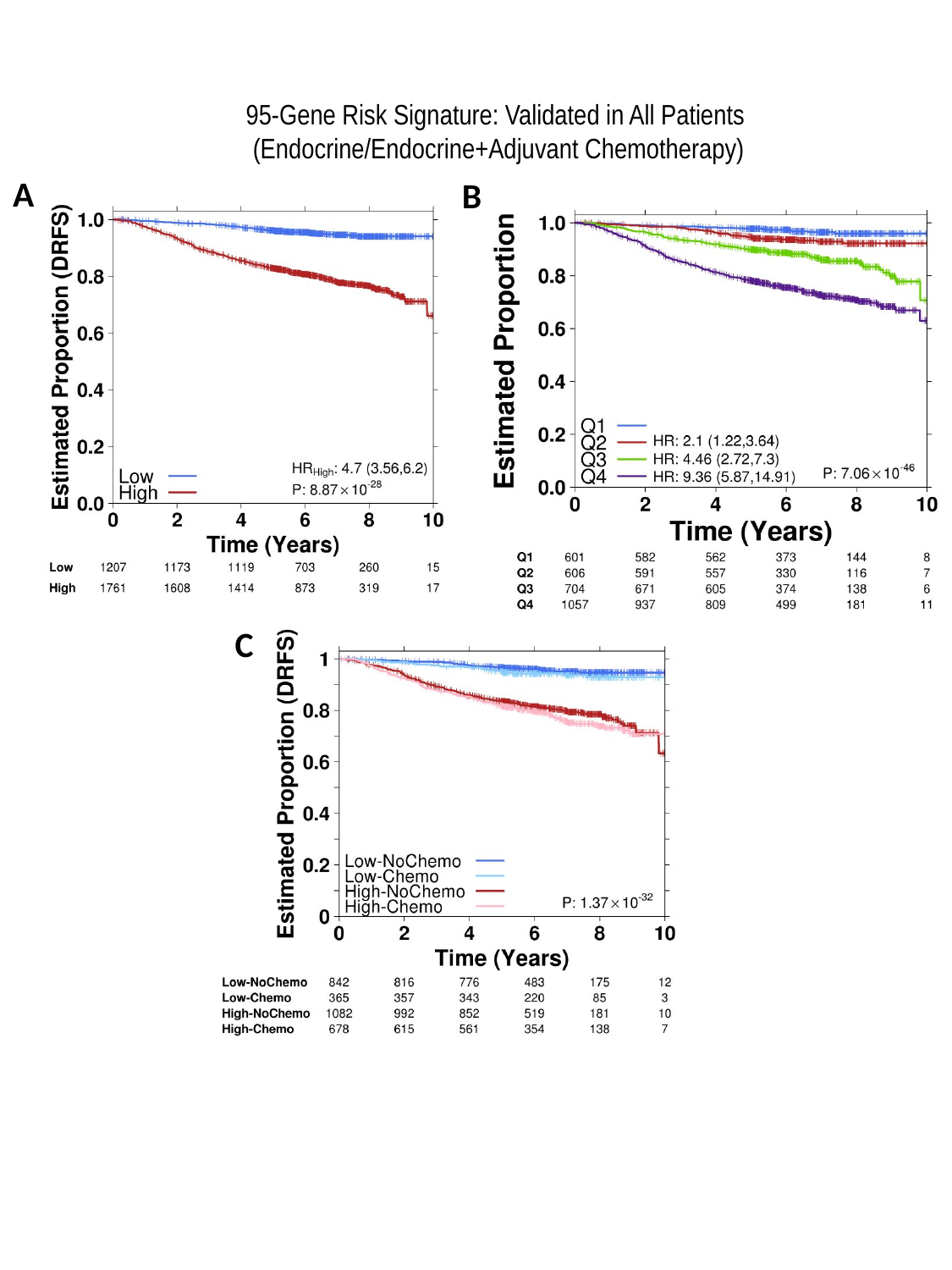

95-Gene Risk Signature: Validated in All Patients
(Endocrine/Endocrine+Adjuvant Chemotherapy)
A
B
C
