# Supplementary material for: Molecular stratification of early breast cancer identifies drug targets to drive stratified medicine
Source: NPJ Breast Cancer. 2017 Feb 15;3:3. doi: 10.1038/s41523-016-0003-5 (PMC5445616; doi:10.1038/s41523-016-0003-5)
Supplement: Supplementary file 7 — Supplementary Figure 6 [file 41523_2016_3_MOESM7_ESM.pptx]

## Slide 1
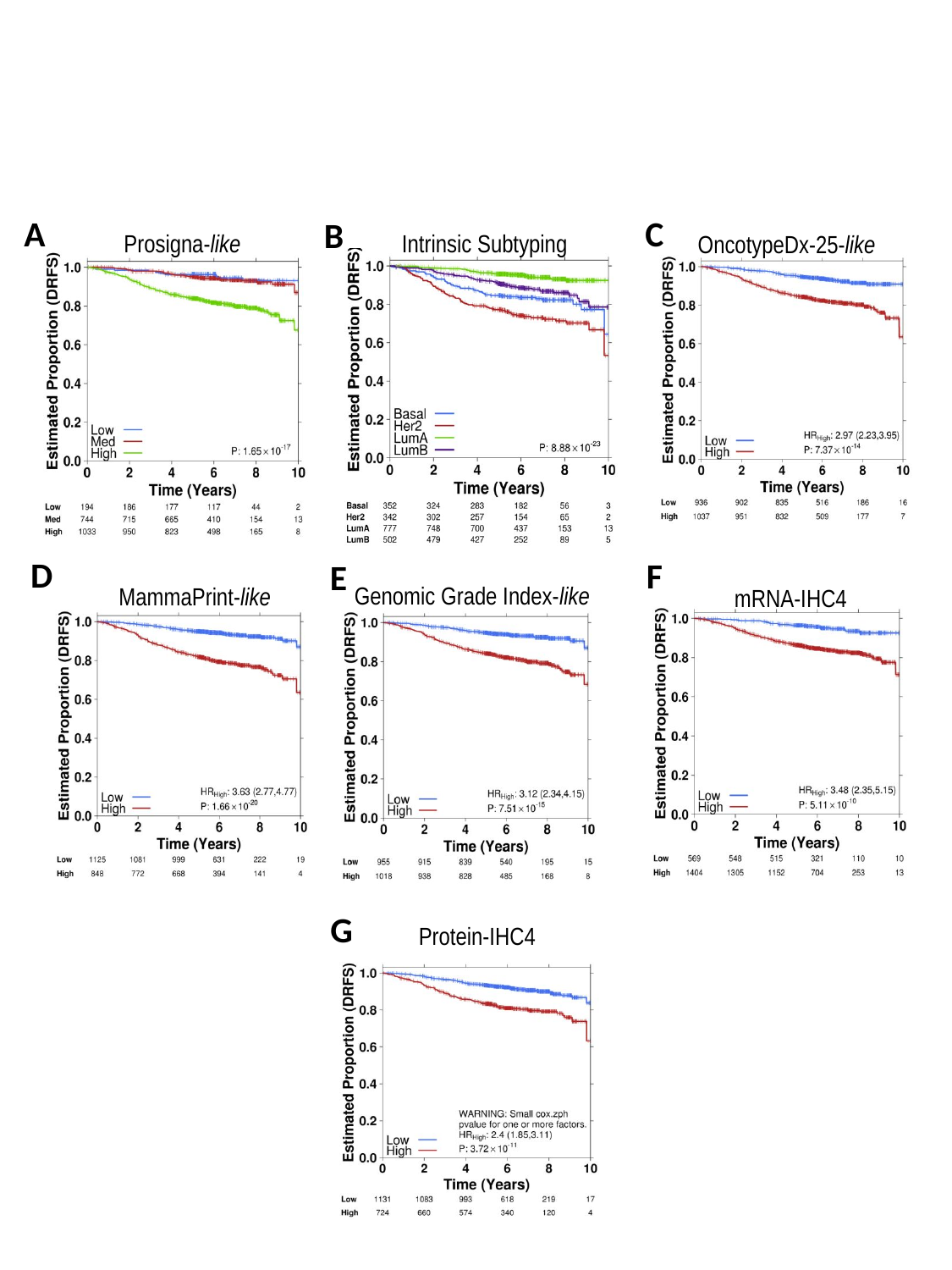

A
C
B
Prosigna-like
Intrinsic Subtyping
OncotypeDx-25-like
D
F
E
Genomic Grade Index-like
MammaPrint-like
mRNA-IHC4
G
Protein-IHC4
