# Supplementary material for: Molecular stratification of early breast cancer identifies drug targets to drive stratified medicine
Source: NPJ Breast Cancer. 2017 Feb 15;3:3. doi: 10.1038/s41523-016-0003-5 (PMC5445616; doi:10.1038/s41523-016-0003-5)
Supplement: Supplementary file 8 — Supplementary Figure 7 [file 41523_2016_3_MOESM8_ESM.pptx]

## Slide 1
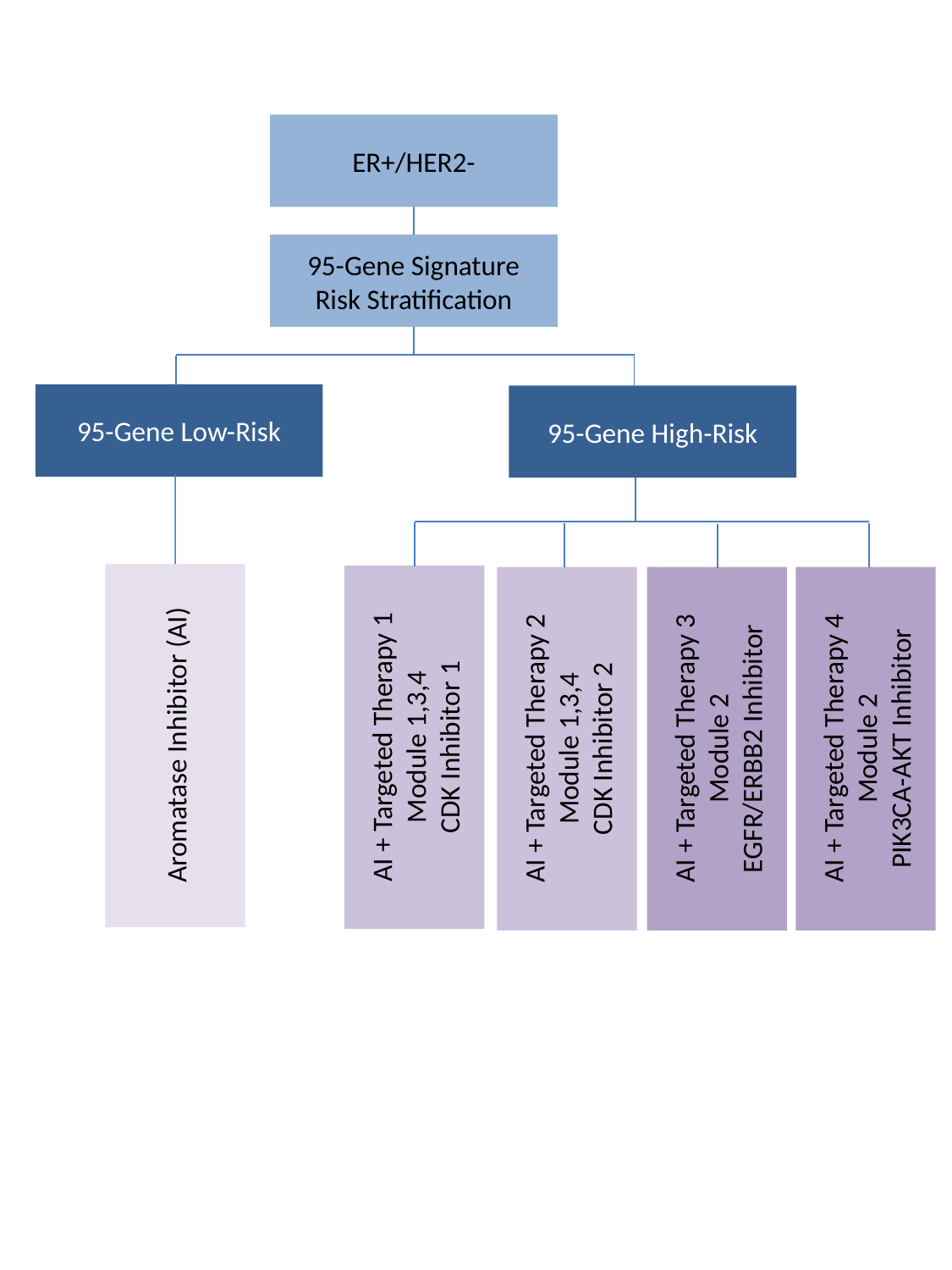

ER+/HER2-
95-Gene Signature Risk Stratification
95-Gene Low-Risk
95-Gene High-Risk
Aromatase Inhibitor (AI)
AI + Targeted Therapy 1 Module 1,3,4
CDK Inhibitor 1
AI + Targeted Therapy 4
Module 2
PIK3CA-AKT Inhibitor
AI + Targeted Therapy 2 Module 1,3,4
CDK Inhibitor 2
AI + Targeted Therapy 3
Module 2
EGFR/ERBB2 Inhibitor
