# Supplementary material for: Molecular stratification of early breast cancer identifies drug targets to drive stratified medicine
Source: NPJ Breast Cancer. 2017 Feb 15;3:3. doi: 10.1038/s41523-016-0003-5 (PMC5445616; doi:10.1038/s41523-016-0003-5)
Supplement: Supplementary file 9 — Supplementary Figure 8 [file 41523_2016_3_MOESM9_ESM.pptx]

## Slide 1
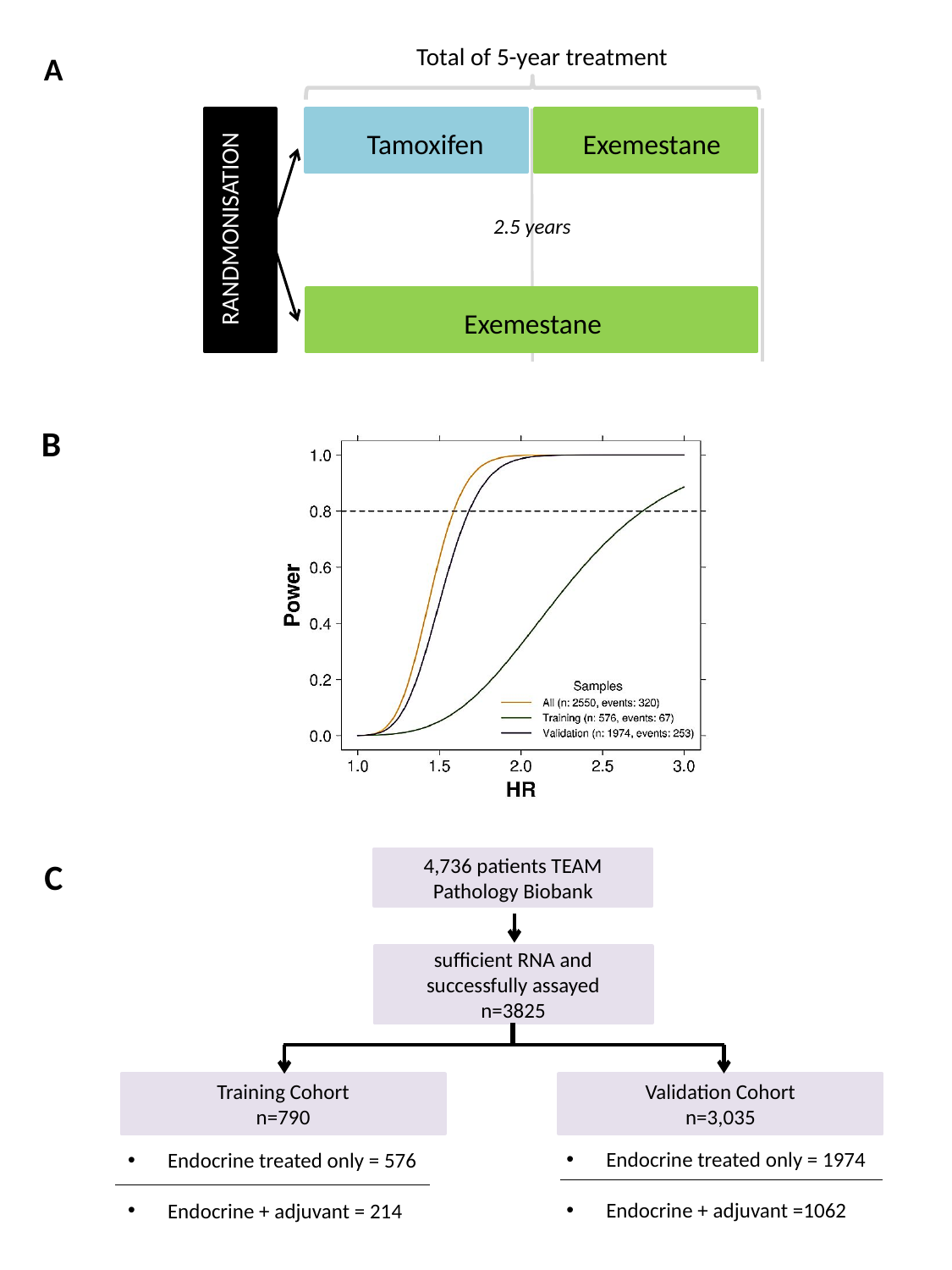

Total of 5-year treatment
Tamoxifen
Exemestane
RANDMONISATION
2.5 years
Exemestane
A
B
C
4,736 patients TEAM Pathology Biobank
sufficient RNA and successfully assayed
n=3825
Training Cohort
n=790
Validation Cohort
n=3,035
Endocrine treated only = 1974
Endocrine + adjuvant =1062
Endocrine treated only = 576
Endocrine + adjuvant = 214
